# ANAPHASE-PROMOTING COMPLEX/CYCLOSOME coactivators maintain AURORA 1 kinase homeostasis during meiotic chromosome segregation

**DOI:** 10.1093/plcell/koaf089

**Published:** 2025-04-17

**Authors:** Jing Xu, Lian Zhou, Kaixin Chen, Runsen Huang, Baixiao Niu, Juanying Ye, Hong Ma, Gregory P Copenhaver, Yingxiang Wang

**Affiliations:** Guangdong Basic Research Center of Excellence for Precise Breeding of Future Crops, Guangdong Laboratory for Lingnan Modern Agriculture, Guangdong Provincial Key Laboratory for the Development Biology and Environmental Adaptation of Agricultural Organisms, College of Life Sciences, South China Agricultural University, Guangzhou 510642, China; School of Biology and Agriculture, Shaoguan University, Shaoguan 512005, China; Guangdong Academy of Agricultural Sciences, Rice Research Institute, Guangzhou 510640, China; Guangdong Basic Research Center of Excellence for Precise Breeding of Future Crops, Guangdong Laboratory for Lingnan Modern Agriculture, Guangdong Provincial Key Laboratory for the Development Biology and Environmental Adaptation of Agricultural Organisms, College of Life Sciences, South China Agricultural University, Guangzhou 510642, China; Guangdong Basic Research Center of Excellence for Precise Breeding of Future Crops, Guangdong Laboratory for Lingnan Modern Agriculture, Guangdong Provincial Key Laboratory for the Development Biology and Environmental Adaptation of Agricultural Organisms, College of Life Sciences, South China Agricultural University, Guangzhou 510642, China; Key Laboratory of Plant Functional Genomics of the Ministry of Education, Jiangsu Key Laboratory of Crop Genetics and Physiology/Co-Innovation Center for Morden Production Technology of Grain Crops, Yangzhou University, Yangzhou 225009, China; National Key Laboratory of Plant Molecular Genetics, CAS Center for Excellence in Molecular Plant Sciences, Institute of Plant Physiology and Ecology, Shanghai Institutes for Biological Sciences, Chinese Academy of Sciences, Shanghai 200032, China; Department of Biology, Eberly College of Science, The Huck Institutes of the Life Sciences, Pennsylvania State University, University Park, PA 16802, USA; Department of Biology and the Integrative Program for Biological and Genome Sciences, University of North Carolina at Chapel Hill, Chapel Hill, NC 27599-3280, USA; Lineberger Comprehensive Cancer Center, University of North Carolina School of Medicine, Chapel Hill, NC 27599-3280, USA; Guangdong Basic Research Center of Excellence for Precise Breeding of Future Crops, Guangdong Laboratory for Lingnan Modern Agriculture, Guangdong Provincial Key Laboratory for the Development Biology and Environmental Adaptation of Agricultural Organisms, College of Life Sciences, South China Agricultural University, Guangzhou 510642, China

## Abstract

Faithful chromosome segregation is essential for both mitotic and meiotic cell division. The anaphase-promoting complex/cyclosome (APC/C) and its coactivators are required for meiotic chromosome segregation, but their potential targets and regulatory mechanisms remain unclear in plants. Here, we performed a ubiquitinome analysis and show that *Arabidopsis thaliana* Aurora 1 (AUR1) is over-ubiquitinated at lysine 102 in the coactivator *Cell Division Cycle 20.1* (*cdc20.1*) mutants and that AUR1 overexpression can partially rescue the *cdc20.1* meiotic defect. We also demonstrate that APC/C ubiquitinates AUR1, leading to its degradation through the 26S proteasome pathway. Moreover, the APC/C subunit and coactivators Cell Cycle Switch 52 A2/B (CCS52A2/B) and CDC20.1 interact with AUR1 both in vitro and in vivo. Intriguingly, CCS52A2/B promotes AUR1 ubiquitination and degradation, while CDC20.1 prevents AUR1 degradation. Consistent with this finding, AUR1 levels are lower in *cdc20.1* and higher in *ccs52* mutants relative to Col-0, and mutation of *CCS52A2*/*B* causes defects in meiotic spindle assembly and homologous chromosome segregation. Genetic analyses demonstrate that Arabidopsis anaphase-promoting complex/cyclosome subunit 8 (APC8), CDC20.1, CCS52 and AUR1 act in the same pathway to control meiotic spindle assembly and homologous chromosome segregation. Thus, this work provides mechanistic insight into the role of APC/C coactivators in regulating AUR1 homeostasis during meiosis in plants.

## Teaser

The ANAPHASE-PROMOTING COMPLEX/CYCLOSOME coactivators CDC20.1 and CCS52s play distinct roles in maintaining AURORA 1 kinase homeostasis during meiotic chromosome segregation in Arabidopsis.

## Introduction

Both mitotic and meiotic cell division require faithful chromosome segregation to ensure appropriate ploidy (Zamariola et al. 2014). Unlike mitosis, meiosis involves 2 rounds of chromosome segregation, with homologous chromosomes (homologs) segregating in meiosis Ⅰ and sister chromatids separating in meiosis Ⅱ to form haploid cells (Zamariola et al. 2014). Defects in meiotic chromosome segregation can cause aneuploidy, which is associated with genome instability and reproductive abnormalities (Chunduri and Storchova 2019). In recent decades, numerous studies have investigated the mechanisms that regulate mitotic chromosome segregation (Cromer et al. 2013), but the regulatory mechanisms controlling meiotic chromosome segregation remain largely elusive. For example, components of the spindle assembly checkpoint (SAC) have been reported to participate in chromosome alignment and segregation during meiosis, but the underlying regulatory mechanisms are unclear, especially in plants.

The SAC senses improper kinetochore-microtubule attachment or abnormal spindle microtubule organization and triggers delay of the cell cycle at metaphase in both mitosis and meiosis (Musacchio and Salmon 2007). The SAC complex contains several highly conserved proteins, including Aurora kinases (Musacchio and Salmon 2007). Aurora homologs belong to a conserved serine/threonine protein kinase family and are critical for proper spindle microtubule assembly and correcting erroneous kinetochore-microtubule attachment, thereby ensuring mitotic and meiotic chromosome separation in multiple species (Demidov et al. 2005; Shrestha et al. 2017; Komaki et al. 2020; Blengini et al. 2021, 2024; Papini et al. 2021; Sen et al. 2021; Ballmer et al. 2024). There are 2 categories of plant Aurora kinases, spindle associated α-Aurora (Aurora 1 and Aurora 2) and the centromere localized β-Aurora (Aurora 3) (Van Damme et al. 2011; Deng et al. 2024a). Single mutants in each exhibit normal cell mitosis, while null alleles of *Aurora aur1-1 aur2-2* double mutants cause embryonic lethality. However, double knockdown *aur1-2 aur2-2* mutants are viable and form abnormal microspore tetrads and micronuclei, but have growth defects (Demidov et al. 2014). Meiosis-specific knockdown of *Aurora* in the flowering plant *Arabidopsis thaliana* causes asynchronous alignment of metaphase chromosomes and unequal segregation at anaphase in meiosis (Niu et al. 2015). In addition, the overaccumulation of Aurora kinases in mouse oocytes and overactivation of Aurora kinases in *Xenopus* egg cells (Ma et al. 2003; Nguyen et al. 2005; Aboelenain and Schindler 2021) or deregulation of Aurora kinases in multiple species (Swain et al. 2008; Jordan et al. 2009; Nikalayevich et al. 2022), including truncated Aurora in Arabidopsis (Demidov et al. 2014; Niu et al. 2015), all cause meiotic SAC dysfunction with increased aneuploidy and decreased fertility. These results support the idea that the Aurora homeostasis is critical for its function, but the underlying regulatory mechanisms are unclear.

The anaphase-promoting complex/cyclosome (APC/C) is a conserved multi-subunit RING E3 ubiquitin ligase that is essential for the ubiquitination and degradation of cell cycle-related substrates (Sivakumar and Gorbsky 2015). Because the APC/C is essential in mitotically dividing cells, null alleles of most APC/C subunits typically cause embryonic lethality (Willems and De Veylder 2022), which prevents the genetic analysis of their effects on postembryonic stages. In yeast and animal mitosis, APC/C facilitates the degradation of Cyclin A and Cyclin B, as well as Sororin and Securin, which promote cohesin removal to facilitate chromosome segregation (Sivakumar and Gorbsky 2015). APC/C also regulates Aurora kinases, Cell Division Cycle 20 (CDC20), and other proteins to control exit from mitosis (Castro et al. 2002; Taguchi et al. 2002). In contrast, in meiosis, the known APC/C substrates are Cyclin B1 and Securin in mouse oocytes (Siomos et al. 2001; Herbert et al. 2003; Rattani et al. 2017), yeast (Buonomo et al. 2000), and Caenorhabditis *elegans* (Siomos et al. 2001); and shugoshin1 (SGO1), monopolar spindle 1 (MPS1), and Cell Division Cycle 20 homolog 1 (Cdh1) in yeast (Jonak et al. 2017). In plant meiosis, genetic analyses suggest that Patronus1 (PANS1) and Rice Salt Sensitive 1 (RSS1), the homologs of Securin in Arabidopsis and rice (Cromer et al. 2019), as well as SWITCH1, the Sororin homolog in Arabidopsis (Yang et al. 2019), are potential APC/C substrates but biochemical or molecular evidence has not been reported.

The catalytic activity of APC/C and its substrate specificity are modulated by evolutionarily and functionally conserved coactivators (Chang et al. 2015), including CDC20 and its homolog Cdh1 (also known as Fizzy-related protein homolog 1 (Fzr1)) in many organisms (Kimata et al. 2008). CDC20 and Cdh1/Fzr1 have been extensively studied in the mitotic cell cycle (Pesin and Orr-Weaver 2008), but their roles in meiosis are not as well characterized. CDC20 is required for meiotic chromosome segregation in *Drosophila melanogaster*, mice and plants (Chu et al. 2001; Jin et al. 2010; Niu et al. 2015), and the yeast and mammalian homologs Cdh1 also participate in meiosis (Blanco et al. 2001; Holt et al. 2011, 2014; Ostapenko and Solomon 2024; Tanno et al. 2020). With the exception of APC/C^Cdh1^, which controls CDK activity in mouse oocytes (Rattani et al. 2017) and the degradation of Cdh1 during meiotic entry in *Saccharomyces cerevisiae* (Ostapenko and Solomon 2024), how coactivators control APC/C activity and specificity in meiosis remains elusive. Moreover, it is not known whether the plant homologous of *Cdh1/Fzr1- Cell Cycle Switch 52* (*CCS52*) are functional in meiosis.

We previously demonstrated that Arabidopsis APC8 and CDC20.1 are required for bivalent alignment and chromosome segregation during meiosis (Niu et al. 2015; Xu et al. 2019), but which substrates APC/C^CDC20.1^ acts on, and how it is regulated are unclear. Here, we identify Aurora 1 (AUR1) as an APC/C substrate. Both in vitro and in vivo analyses show that APC/C mediates AUR1 ubiquitination and degradation by the 26S proteasome pathway. Interestingly, although both CDC20.1 and CCS52A2/B coactivators interact with AUR1, they play opposite roles in inhibiting and promoting AUR1 degradation. Consistent with these findings, CCS52A2 and CCS52B have partially redundant functions in the alignment and segregation of meiotic bivalents as APC/C and CDC20.1. The overaccumulation of AUR1 in *ccs52* mutants causes meiotic defects similar to those in *apc8-1, cdc20.1*, and *ProDMC1:Aurora1^RNAi^*. Taken together, our results provide mechanistic insight into how APC/C and coactivators coordinately regulate AUR1 homeostasis during meiosis in plants. Because APC/C, its coactivators and Aurora kinase are highly conserved in structure and function among eukaryotes, we speculate that this mechanism for regulating Aurora kinase stability to ensure faithful meiotic chromosome segregation might be shared in other organisms.

## Results

### Identification and validation of the AUR1 ubiquitination

To identify the potential targets of APC/C^CDC20.1^ during meiosis (Niu et al. 2015), we performed ubiquitin (ub)-modified proteomic analysis by immunoprecipitation (IP)-mass spectrometry (MS)/MS using young inflorescences of wild type (WT, Col-0) and *cdc20.1-3* mutant plants. We identified 1,260 ubiquitinated proteins, of which 1,031 showed no obvious alternation of ubiquitination level. Proteins with a >1.5-fold change in ubiquitination level between *cdc20.1-3* and Col-0 (*P*-value < 0.05) were considered differentially ubiquitinated proteins, 229 proteins were differentially ubiquitinated in *cdc20.1-3* compared with Col-0 with 207 of them having higher ubiquitination levels and only 22 proteins having lower (Supplementary Table S1). Gene ontology (GO) annotations for the 207 proteins found significant enrichment in chromosome organization and protein metabolism (Fisher's exact test, *P*-value < 0.05) (Supplementary Fig. S1A). Interestingly, AUR1 is included in both processes, and its ubiquitination level at lysine 102 (K102) located in its kinase domain was 3.6-fold higher in *cdc20.1* compared with Col-0 (Supplementary Fig. S1B), indicating that CDC20.1 negatively regulates AUR1 ubiquitination. AUR1 is a conserved Ser/Thr protein kinase with a kinase domain that occupies almost the entire protein, and a conserved destruction box (D-box) domain at its C terminus (Supplementary Fig. S1C). We constructed an intact AUR1 expression construct, and mutant construct AUR1K102A in which K102 is substituted by alanine (Supplementary Fig. S1C). To test whether AUR1 can be ubiquitinated in vivo, we transiently expressed an AUR1-GFP fusion construct in *Nicotiana benthamiana* leaves treated with MG132 (a chemical inhibitor of the 26S proteasome) followed by IP with an anti-GFP antibody crosslinked to magnetic beads. Western blots probed with anti-GFP antibody had a smear of signal above the main AUR1 band that may correspond to ubiquitinated forms in contrast to the GFP negative control, consistent with the results obtained using an anti-UBQ11 (polyubiquitin) antibody (Supplementary Fig. S1D), suggesting that AUR1 is ubiquitinated in vivo. To exclude the possibility of nonspecific absorption by the magnetic beads, we validated these results using Tandem-repeated Ubiquitin-binding Entities (TUBE2) affinity gel matrix, which specifically binds polyubiquitinated proteins (Hjerpe et al. 2009). Western blots of transiently expressed AUR1-GFP, and AUR1K102A-GFP and GFP controls probed with anti-GFP or anti-UBQ11 had a smear of signal indicative of ubiquitinated AUR1 and AUR1K102A in both GFP magnetic-bead and TUBE2 processed samples (Supplementary Fig. S1E). However, AUR1K102A showed attenuation of the ubiquitinated band compared with AUR1 in the TUBE2 processed samples (Supplementary Fig. S1E), indicating that mutation of K102 reduces AUR1 ubiquitination levels.

**Figure 1.**
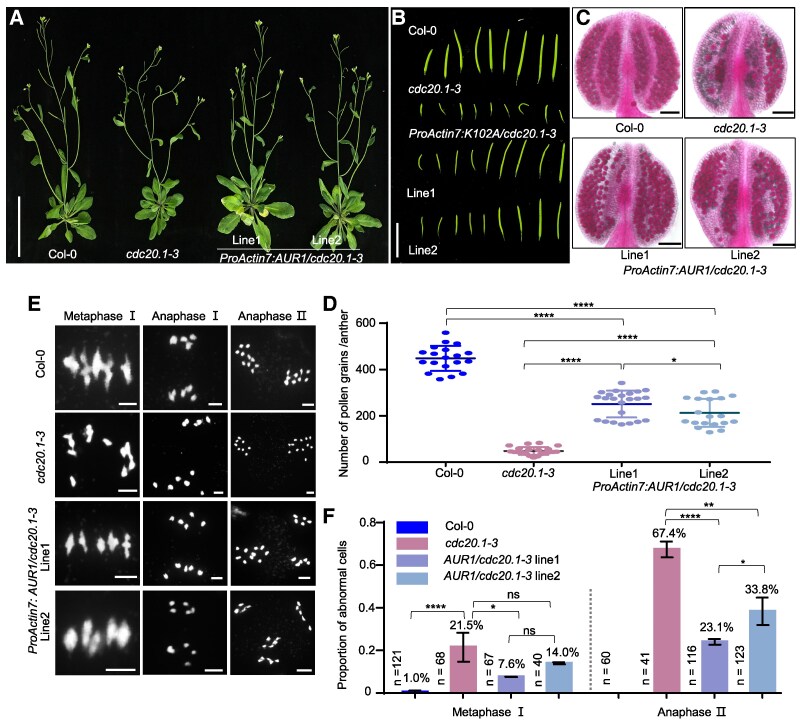
AUR1 is required for fertility and meiosis in *cdc20.1-3*. **A)** to **(C)** Whole plants **(A)**, the first 9 siliques **(B)** and pollen grains stained with Alexander Red **(C)** of Col-0, *cdc20.1-3*, and 2 independent lines of *ProActin7:AUR1-FLAG/cdc20.1-3* plants. Scale bar, 5 cm **(A)**, 1 cm **(B)**, 100 *μ*m **(C)**. **D)** Quantification of viable pollen grains per anther from Col-0 (*n* = 19), *cdc20.1-3* (*n* = 21), and *ProActin7:AUR1/cdc20.1-3* complemented plants with line 1 (*n* = 23) and line 2 (*n* = 19). Data are presented as means ± SD. ****P < 0.0001, *P < 0.05. Statistical analysis was performed using 2-sided 2-tailed Student's *t* test. **E)** Meiotic chromosome morphology of Col-0, *cdc20.1-3*, and *ProActin7:AUR1/cdc20.1-3* complemented Arabidopsis. Scale bar, 5 *μ*m. **F)** Histogram showing the proportion of cells with meiotic defects from Col-0, *cdc20.1-3*, *ProActin7:AUR1-FLAG/cdc20.1-3* Arabidopsis plants at metaphase Ⅰ and anaphase Ⅱ in **(E)**. For these meiotic stages, cells isolated from >3 independent plants were observed. The numbers and proportions near the bar indicate the number of cells counted and the percentage of abnormal meiocytes. Data are presented as means ± SD. *P < 0.05, **P < 0.01, ****P < 0.0001, ns, no significance, statistical analysis was performed using 2-tailed Fisher's exact test. Source data are provided as a Supplementary data set S1.

To investigate whether AUR1 was related to the meiotic defects in *cdc20.1* in vivo, we transformed AUR1-FLAG driven by the well-characterized *Actin7* promoter in meiocytes (Wang et al. 2022, 2023; Xu et al. 2023) into *cdc20.1-3* heterozygous mutants and selected independent lines for cytological experiments (Supplementary Fig. S1F). *ProActin7:AUR1-FLAG/cdc20.1* transgenic plants had vegetative phenotypes similar to *cdc20.1* and Col-0 (Fig. 1A). Mutant *cdc20.1-3* plants have strong fertility defects including under-developed siliques, inviable pollen, and abnormal chromosome segregation as previously reported (Niu et al. 2015) (Fig. 1, B to E). Transgene expression of *AUR1* partially rescues these *cdc20.1-3* phenotypes (Fig. 1, B and C). Pollen grain viability measured by Alexander Red staining in *ProActin7:AUR1-FLAG/cdc20.1* (251 ± 12/anther, *n* = 23, line1; 213 ± 14/anther, *n* = 19, line2) is significantly higher compared with *cdc20.1-3* (48 ± 3, *n* = 21) (Student's *t* test, ****P < 0.0001) (Fig. 1D). Moreover, male meiocyte chromosome spreads stained with 4′,6′-diamidino-2-phenylindole (DAPI) from *ProActin7:AUR1-FLAG/cdc20.1* transgenic plants have lower frequencies of meiotic defects including chromosome misalignment at metaphase (rate ratio, 7.6%; 95% CI, 3.3% to 16.5% in line 1; rate ratio, 14.0%; 95% CI, 7.0% to 26.2% in line2) and unequal segregation at anaphase (rate ratio, 23.3%; 95% CI, 16.5% to 31.7% in line 1; rate ratio, 37.4%; 95% CI, 29.4% to 46.2% in line2) compared with *cdc20.1* (Figure 1, E and F). Statistical analysis of meiotic defects in individual stages showed that transgene expression of AUR1 is able to partially rescue the *cdc20.1* meiotic defects, suggesting that the AUR1 is required for meiosis in *cdc20.1* mutant.

### Expression of AUR1K102A causes a meiotic chromosome segregation defect

Expression of truncated AUR1 (including a small part of the kinase domain but without D-box) causes higher rates of meiotic abnormalities and aneuploidy in Arabidopsis (Demidov et al. 2014). To test the effect of *AUR1K102A* in meiosis, we generated transgenic plants overexpressing either intact *AUR1* or *AUR1K102A* under the control of *HTR2*, *DMC1*, and *CDC20.1* promoters in the Arabidopsis Col-0 background. We selected 2 independent transgenic lines of each construct for verification of AUR1K102A protein expression (Supplementary Fig. S2A) and phenotypic observation. Toluidine blue stained tetrad stage meiocytes in the *AUR1K102A*-overexpressing plants showed polyads or atypical microspore tetrads, indicative of abnormal meiosis, while no or few polyads were observed in Col-0 or *AUR1*-overexpressing plants (Supplementary Fig. S2B). We examined meiotic chromosome morphology using FISH and found that *AUR1K102A* plants have unbalanced chromosome segregation (Supplementary Fig. S2C), similar to previous reports (Demidov et al. 2014). In contrast, the *AUR1-*overexpressing plants have chromosome morphologies that are indistinguishable from Col-0 (Supplementary Fig. S2C), which may be due to the plant's native ubiquitination degradation mechanism (Supplementary Fig. S2A), while overexpressing *AUR1K102A* causes meiotic defects (Supplementary Fig. S2C), supporting the idea that the *AUR1K102* ubiquitination plays an important function in meiosis.

### APC/C facilitates the ubiquitination of AUR1 for degradation

**Figure 2.**
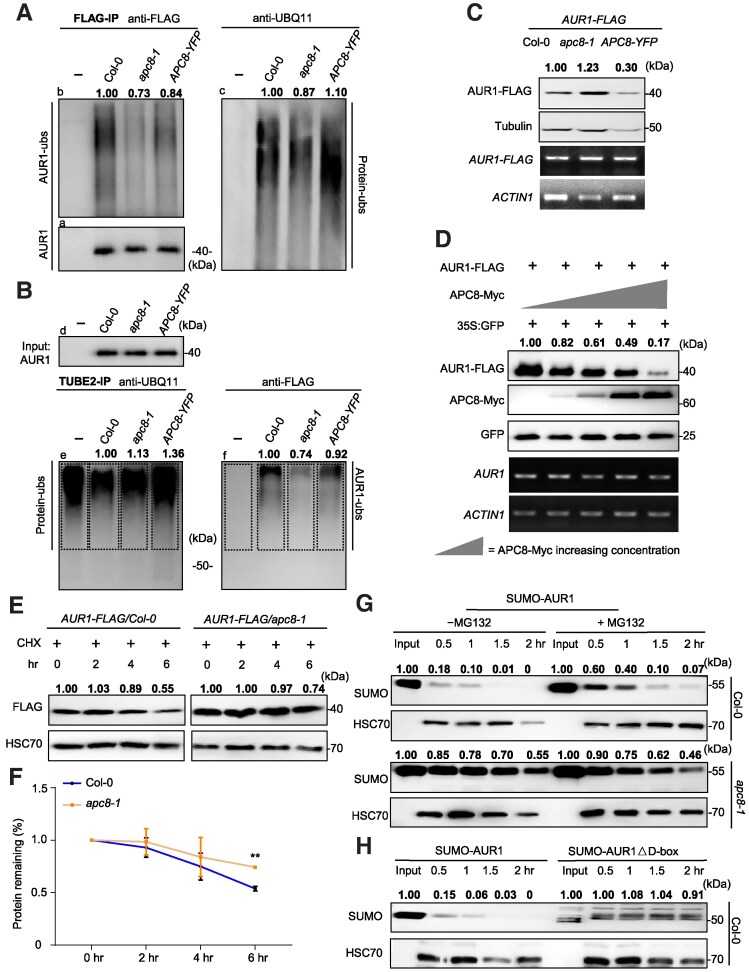
APC/C mediates the ubiquitination and degradation of AUR1 both in vitro and in vivo. **A)** to **(B)** AUR1 ubiquitination is associated with APC/C. Total proteins of central inflorescences from *ProActin7-FLAG/Col-0* (control)*, ProActin7:AUR1-FLAG/Col-0, ProActin7:AUR1-FLAG/apc8-1, ProActin7:AUR1-FLAG/APC8-YFP* (designated as “-, Col-0, *apc8-1*, *APC8-YFP*,” respectively) treated with 50 *μ*M MG132 were extracted and immunoprecipitated with anti-FLAG **(A)** and TUBE2 affinity gel matrix **(B)**. **A)** Anti-FLAG antibody showed similar AUR1 protein (bottom, left, a) in IP samples, and AUR1 ubiquitination level above AUR1 target band was compared using anti-FLAG (top, left, b) and UBQ11 (right, c) antibodies. Anti-FLAG antibody (d) in **(B)** showed equal AUR1 protein for TUBE2-IP assay, IP products were detected by anti-UBQ11 and anti-FLAG to detect ubiquitinated proteins (e) and ubiquitinated AUR1 (f). Representative immunoblot results with the relative smear ladders intensities in (A-c) normalized to (A-a), and relative smear ladders intensities in (B-f) normalized to Input (B-d) were labeled. The value of the samples with Col-0 background was set as 1.0. Three independent experiments were performed with similar results (100 inflorescences from 25 plants in each replicate at the same treated time). **C)** Immunoblotting detection of protein level of AUR1 in Col-0, *apc8-1* and *APC8-YFP* backgrounds. Anti-FLAG and anti-Tubulin antibodies show the AUR1 protein and loading control (top 2), respectively. Target gene *AUR1* and house-keeping gene *ACTIN1* mRNA expression levels were analyzed (bottom 2 panels). The value of samples with Col-0 background was set as 1.0. Representative immunoblot results with the relative band intensities of AUR1-FLAG normalized to Tubulin were labeled. Four independent experiments were performed with similar tendency (50 central inflorescences from 15 plants in each replicate at the same treated time). **D)** In vivo degradation assay shows that APC8 promotes AUR1 protein instability. Anti-FLAG and anti-Myc antibodies were used to detect the amount of AUR1 and APC8 (top 2). Relative band intensities of AUR1 normalized to reference protein GFP (middle) were labeled. The value of samples without expressing APC8-Myc was set as 1.0. Total RNA was extracted from the injected *N. benthamiana* leaves, RT-qPCR of *AUR1* and *ACTIN1* determines the transcript level (bottom panels). Three biological replicates were performed with similar results (6 *N. benthamiana* strains in each replicate at the same treated time). **E** and **F)** AUR1 shows a lower turnover rate in *apc8-1* compared with Col-0 in Arabidopsis using in vivo degradation assay in **(E)**, with quantitative analysis in **(F)**. The protein extracts of inflorescences were treated with 100 mm translation inhibitor CHX and incubated at 4 ℃ for indicated time periods, followed by immunoblotting using antibody against FLAG (top), HSC70 was used as the loading control (bottom). Relative band intensities of AUR1-FLAG normalized to loading control protein were labeled. The value of samples in original state (0 h, 0 h) was set as 1.0 in **(E)**. The level of AUR1 was plotted based on the level at 0 h (1.0). Three independent biological repeats were performed with similar tendency (30 central inflorescences from 15 plants in each replicate at the same treated time). The error bar represents SD of test. **P < 0.01, statistical analysis was performed using 2-tailed Student's *t* test in **(F)**. **G)** AUR1 degrades faster in Col-0 compared with *apc8-1* in cell-free degradation assay. HSC70 was used as the loading control to indicate the protein loaded (second and bottom panel). **H)** The decay rate of AUR1 is higher in intact AUR1 compared with AUR1△D-box in cell-free degradation assay. HSC70 antibody was used to determine the loading sample (bottom). In **(G)** to **(H)**, representative immunoblot results with the relative band intensities of SUMO-AUR1 normalized to loading control protein were labeled. The value of input protein was set as 1.0. and experiments were conducted in 3 replicates with similar results (100 inflorescences from 30 Col-0 and 60 *apc8* plants in each replicate at the same treated time). Source data are provided as a Supplementary data set1 and supported by Supplementary Figs. S3 and S4.

As a coactivator, CDC20.1 binds APC8 and APC3 subunits to trigger APC/C E3 ligase catalytic activity, which enables ubiquitin chain transfer to substrates (Kimata et al. 2008). We previously obtained a meiosis-specific knockdown of *AUR1* (*ProDMC1:Aurora1^RNAi^*, designated as *AUR^RNAi^*) (Niu et al. 2015), which has defects in meiotic chromosome alignment and segregation similar to *apc8-1* and *cdc20.1-3* (Niu et al. 2015; Xu et al. 2019), indicating that AUR1 may be a target of APC/C^CDC20.1^. To test whether APC/C mediates AUR1 ubiquitination, we conducted in vivo ubiquitination assays using stable transgenic plants expressing AUR1-FLAG in Col-0 and *apc8-1* backgrounds, as well as APC8-overexpression (*APC8-YFP*) lines (Zhong et al. 2019). IP using anti-FLAG agarose beads showed that, in contrast to negative controls, smeared ladders of AUR1 (indicative of ubiquitinated proteins) are weaker in *apc8-1* compared with Col-0 (Student's *t* test, ***P = 0.0008) and *APC8-YFP* plants (Student's *t* test, **P = 0.0069) (Fig. 2A and Supplementary Fig. S3A), showing that AUR1 ubiquitination levels are positively associated with APC/C activity. Consistent with these observations, we incubated total protein extracts containing approximately equal amounts of AUR1 with TUBE2 beads to compare the extent of AUR1 ubiquitination after enrichment for ubiquitinated proteins, and found that AUR1 ladders are more intense in Col-0 compared with *apc8-1* (Student's *t* test, *P = 0.0101) (Fig. 2B and Supplementary Fig. S3B), indicating that mutation of APC8 decreases AUR1 ubiquitination levels. In contrast, AUR1 ubiquitination levels in APC8-overexpressing plants are indistinguishable compared with Col-0 (Student's *t* test, *P* = 0.9752 in 2A, *P* = 0.054 in 2B) (Fig. 2, A to B, and Supplementary Fig. S3, A and B), indicating that AUR1 in Col-0 may be close to maximal levels of ubiquitination or that over-ubiquitinated AUR1 may be rapidly degraded in APC8-overexpressing plants. To validate these observations, we used an in vitro ubiquitination assay to show that AUR1 ubiquitination is positively regulated by APC/C when recombinant AUR1 from *Escherichia coli* is incubated with lysates from APC8-overexpressing plants, which results in more intense ubiquitination, and *apc8-1* which results in less ubiquitination (Supplementary Fig. S4A). Together, both in vivo and in vitro ubiquitination assays support the idea that APC/C catalyzes the polyubiquitination of AUR1.

Polyubiquitination usually associates with protein degradation (Meyer and Rape 2014). To test whether APC/C-mediated polyubiquitination of AUR1 is related to degradation, we examined AUR1 levels in protein extracts from inflorescence of Col-0, transgenic and mutant plants and observed significantly higher (Student's *t* test, *P = 0.0196) and lower (Student's *t* test, ****P < 0.0001) AUR1 levels in *apc8-1* and APC8-overexpressing plants compared with Col-0, respectively (Fig. 2C and Supplementary Fig. S3C). *ProActin7:AUR1-FLAG* transcript levels are equivalent in the corresponding lines (Fig. 2C), implying that APC/C affects AUR1 protein stability but not mRNA abundance. We validated these observations using an independent in vivo degradation assay with proteins transiently expressed in *N. benthamiana* leaves and found that AUR1 levels decrease with increasing APC8 levels (Fig. 2D, Supplementary Fig. S3D and Supplementary data set S1). We used a similar transient expression degradation assay to show that APC8 promotes AUR1 degradation in a time-dependent manner (Supplementary Fig. S4, C and D). Furthermore, western blots of proteins extracted from Arabidopsis inflorescences over a time course treated with cycloheximide (CHX), an inhibitor of eukaryotic protein synthesis, show that the rate of AUR1 degradation is decreased in *apc8-1* compared with Col-0 (Fig. 2, E and F). Moreover, using an in vitro cell-free degradation assay, we show that the decay rate of AUR1 is higher in Col-0 compared with *apc8-1* at all-time points (Student's *t* test, *P = 0.0205) (Fig. 2G and Supplementary Fig. S3E), suggesting that APC/C mediates the degradation of AUR1 protein.

Polyubiquitin is formed by the linkage of individual ubiquitin units through lysine residues and homotypic chains linked by lys-11 or lys-48, generated by APC/C, can be recognized by the 26S proteasome (Meyer and Rape 2014). To investigate whether AUR1 ubiquitination leads to proteasomal degradation, we used in vivo transient expression assay in *N. benthamiana* leaf by conjunction of 26S proteasome inhibitor MG132. AUR1 (Student's *t* test, ***P = 0.0007) and AUR1K102A (Student's *t* test, *P = 0.0267) levels were significantly elevated in leaves treated with MG132 compared with untreated leaves, but AUR1K102A was less sensitive to MG132 (Student's *t* test, ns = 0.0679) (Supplementary Fig. S4B and Supplementary data set S1), consistent with its low ubiquitination levels (Supplementary Fig. S1E). In addition, treatment with 50 *μ*M MG132 has a significant effect in blocking the degradation of AUR1 in the presence of APC8 (Student's *t* test, *P = 0.0274) (Supplementary Fig. S4, E and F). Similar results were observed using the cell-free degradation assay, in which AUR1 protein stability was enhanced with the addition of MG132 when incubated with Col-0 (Student's *t* test, ns = 0.086) and *apc8-1* lysate (Student's *t* test, *P = 0.0462) (Fig. 2G and Supplementary Fig. S3E). Taken together, these data provide strong evidence that APC/C negatively regulate AUR1 stability by mediating its ubiquitination for processing by the 26S proteasomal degradation pathway.

### APC/C subunit APC10 recognizes the AUR1 D-box both in vitro and in vivo

**Figure 3.**
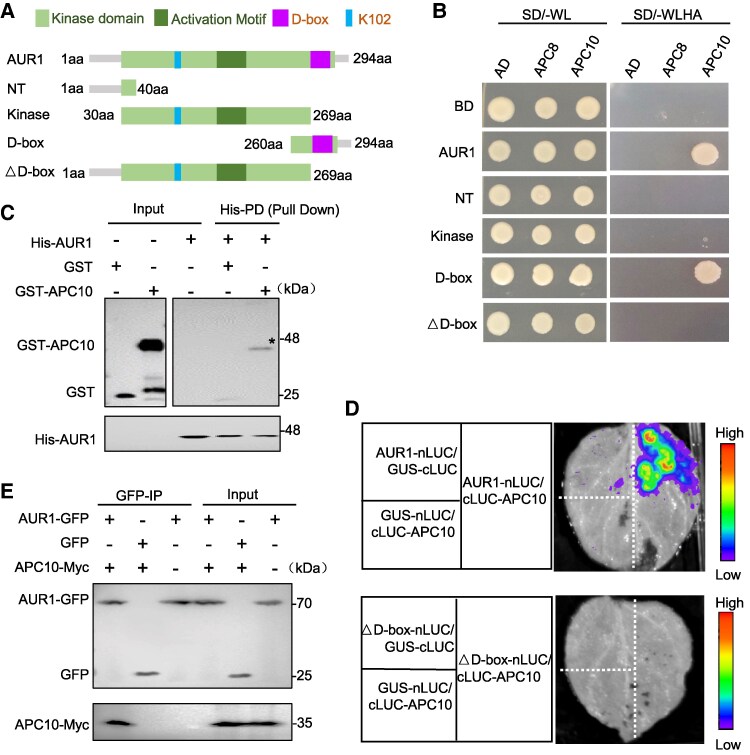
AUR1 interacts with APC10 both in vitro and in vivo. **A)** Schematic diagram of full-length AUR1 and various deletion derivatives used in the following analysis. Numbers refer to the positions of the first or last amino acid in the sequences. The rectangle boxes with various colors indicate the domain organizations of AUR1. Ubiquitinated residue in AUR1 is shown. **B)** Analysis of the interaction between AUR1 and APC10 using yeast Y2H system. *pGADT7 (AD)* and *pGBKT7 (BD)* empty vectors are used as negative control. Transformants were plated on synthetic dropout (SD) medium without leucine or tryptophan (SD/-WL), synthetic dropout (SD) medium alanine (SD/-WLHA) to detect interactions. **C)** In vitro pull-down assay examines the interaction between AUR1 and APC10, whose protein are purified from expression in *E. coli* (His-AUR1, GST, GST-APC10). His-AUR1 proteins immobilized on Ni-NTA His-bind affinity beads were incubated with GST or GST-APC10. Samples were analyzed by immunoblotting with anti-GST (top) and anti-His antibodies (bottom). Input proteins and pull-down proteins are shown in the first and second columns. The asterisk (*) indicate the target protein location. **D)** split-luciferase complementation assay examines the interaction between AUR1, truncated AUR1 (△D-box) and APC10 in *N. benthamiana* leaves. AUR1 and AUR1△D-box were fused with N-terminal fragments of Luciferase (nLUC), APC10 was fused with cLUC. The color legend (right) indicates the fluorescence intensity. GUS-nLUC, GUS-cLUC were used as negative controls. **E)** Co-IP assay shows that AUR1 interacts with APC10 in vivo. GFP-tagged AUR1 was co-expressed with Myc-tagged APC10 in *N. benthamiana* leaves and using GFP and APC10-Myc co-expression line as negative control. Three independent replicates showed the same results in B to E.

The APC/C subunit APC10 along with APC/C coactivators act as substrate co-receptors that bind the D-box of substrates to mediate their ubiquitination (da Fonseca et al. 2011). The C terminus of AUR1 has a conserved D-box (Supplementary Fig. S1C), so we hypothesized that the AUR1 D-box may be recognized by APC/C. To test their possible interaction, we used a yeast 2-hybrid (Y2H) assay with full-length AUR1 and various deletion derivatives as bait (Fig. 3A). AUR1 interacts with APC10 specifically but not with APC8 (Fig. 3B) and the D-box domain was sufficient for the interaction, while other truncated forms lacking D-box did not interact (Fig. 3B). We confirmed the interaction using a pull-down assay to show that the recombinant His-AUR1 protein is precipitated by GST-APC10 but not GST alone (Fig. 3C). To validate the interaction of APC10 and AUR1 in vivo, we used a Split-Luciferase Complementation assay with APC10 and AUR1 fused to C-terminal fragment of Luciferase (cLUC) and N-terminal fragment of Luciferase (nLUC), respectively. Strong luciferase activity (LUC) was observed in *N. benthamiana* leaves when co-expressed APC10 and AUR1, while no detectable LUC signal was observed with APC10 and truncated AUR1 lacking its D-box (AUR1△D-box) or in negative controls (Fig. 3D). As an independent validation, we used a co-immunoprecipitation (Co-IP) assay with AUR1 and APC10 transiently co-expressed in *N. benthamiana* leaves to show that AUR1-GFP is coprecipitated with APC10-Myc while GFP alone is not (Fig. 3E). Together, these results demonstrated that AUR1 interacts with APC10 in a D-box-dependent manner. Consistent with these observations, the AUR1△D-box is obviously stable compared with the intact AUR1 in the cell-free degradation assay (Fig. 2H, Supplementary Fig. S3F and Supplementary data set S1), which strengthens the conclusion that APC/C binds and mediates the degradation of AUR1 by recognizing its D-box.

### APC/C coactivators CCS52A2/B but not CDC20.1 promote AUR1 degradation

**Figure 4.**
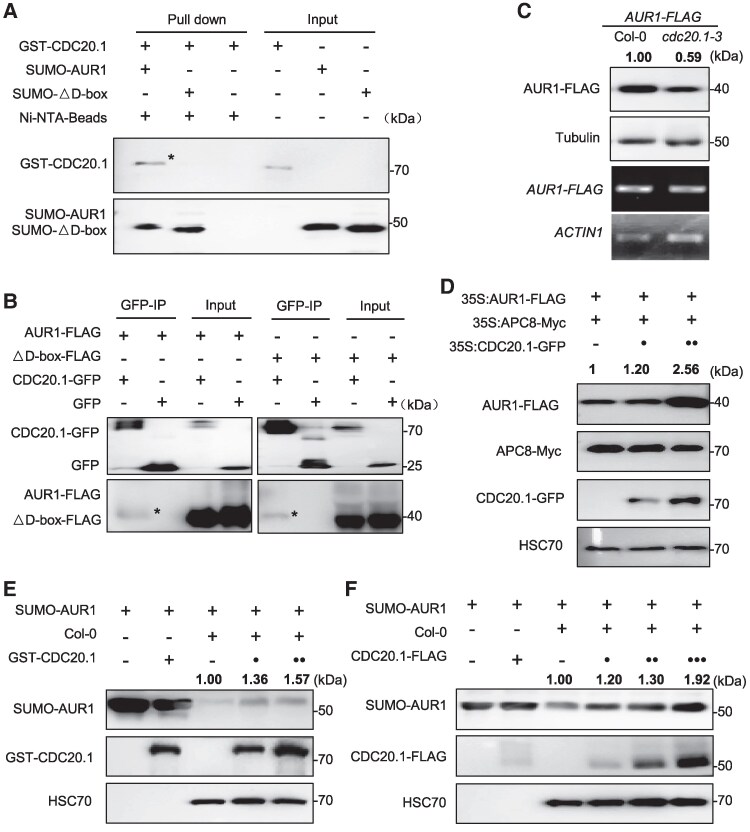
CDC20.1 interacts with AUR1, but is dispersible for its degradation. **A)** In vitro pull-down assay shows the interaction between AUR1, truncated protein AUR1△D-box and CDC20.1. The mixtures of the purified proteins GST-CDC20.1 were pulled down by SUMO-AUR1 and SUMO-△D-box immobilized on the Ni-NTA affinity agarose beads and analyzed by immunoblots with anti-GST antibody (top) and anti-SUMO antiserum (bottom). The asterisk (*) indicates the target band. **B)** Co-IP assay shows interaction of AUR1, truncated protein AUR1△D-box with CDC20.1 in vivo. FLAG-tagged AUR1 was co-expressed with GFP-tagged CDC20.1 in *N. benthamiana* leaves. The asterisks (*) indicate the target bands. **C)** AUR1 protein in inflorescences was decreased in *cdc20.1-3* relative to Col-0. Tubulin is the loading control. The results of 3 biological replicates show similar tendency (50 central inflorescences from 20 plants in each replicate treated at the same time). The ratio of the relative density between *cdc20.1*-3 and Col-0 signals (*cdc20.1-3*/Col-0) in the representative immunoblot result were labeled. mRNA expression levels of the target gene *AUR1* and house-keeping gene *ACTIN1* were analyzed (bottom 2 panels). **D)** In vivo degradation assay shows the role of CDC20.1 in protecting AUR1 stability. HSC70 was the internal control (bottom). The value of samples without expressing coactivators was set as 1.0. **E)** to **(F)** Cell-free decay assay supports the role of CDC20.1 in AUR1 stability. SUMO-AUR1 was incubated with equal amount of Col-0 lysate with an extra addition of CDC20.1 expressed in *E. coli* in **(E)** and immunoprecipitated products from inflorescences of transgenic plant using antibodies against FLAG in **(F)**. Immunoblot was performed with anti-SUMO antiserum (top), anti-GST or anti-FLAG antibodies (middle). HSC70 was the internal control (bottom). Source data are provided as a Supplementary data set S1 and supported by Supplementary Fig. S5A-, to C.

APC/C coactivators participate in substrate recognition, binding, and activation of APC/C activity, to promote substrate ubiquitination and degradation (Chang et al. 2015). Our results showed that CDC20.1 deficiency results in the increase of AUR1 ubiquitination levels (Supplementary Fig. S1B), suggesting that CDC20.1 does not promote AUR1 ubiquitination and degradation. To test this hypothesis, we examined whether CDC20.1 interacts with AUR1. Using an in vitro pull-down assay, we show that CDC20.1 interacts with AUR1 (Fig. 4A). However, an in vivo Co-IP assay shows that they interact in a D-box-independent manner (Fig. 4B), suggesting the existence of other bridging factors that can facilitate their interaction in vivo. Western blot analysis shows that AUR1 protein levels were lower in *cdc20.1-3* relative to Col-0 using central inflorescence of stable *AUR1-FLAG* transgenic Arabidopsis plants (Student's *t* test, ****P < 0.0001) (Fig. 4C and Supplementary Fig. S5A), suggesting that CDC20.1 stabilizes AUR1 rather than promotes its degradation. To test this idea, we used an in vivo degradation assay using recombinant AUR1 transiently expressed in *N. benthamiana* and found that APC/C-mediated degradation of AUR1 is inhibited with increasing levels of CDC20.1 (Figure 4D, Supplementary Fig. S5B and Supplementary data set S1). We also validated these results using a cell-free degradation assay (Fig. 4, E to F and Supplementary Fig. S5C). The anticorrelation between AUR1 degradation and increasing CDC20.1 protein level suggests that CDC20.1 promotes AUR1 stabilization instead of degradation.

**Figure 5.**
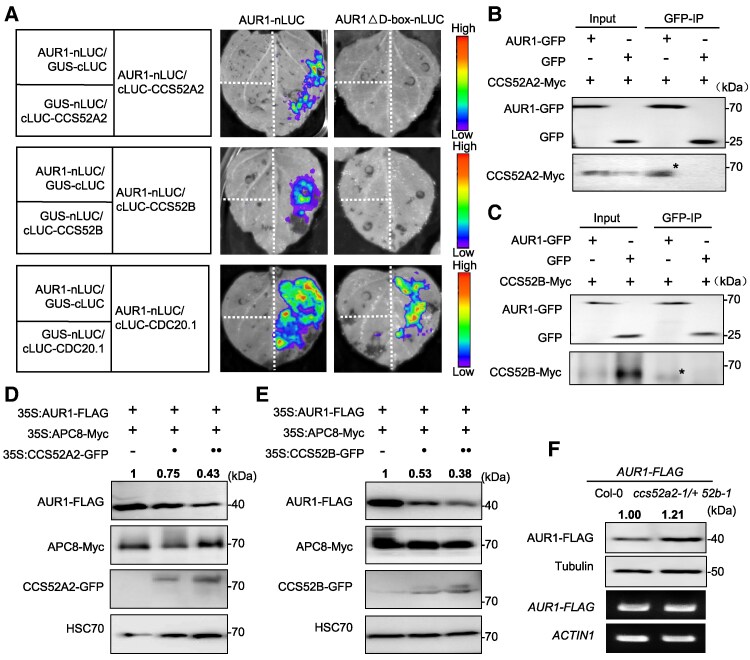
CCS52s interact with AUR1 and promote its degradation. **A)** Split-luciferase complementation assay shows AUR1 interacting with CCS52 and CDC20.1 in a D-box-dependent or -independent manner in *N. benthamiana* leaves. AUR1 and AUR1△D-box were fused with nLUC, coactivators were fused with cLUC. The color legend on the right indicates the florescence signal intensity (right). GUS-nLUC, GUS-cLUC were used as negative controls. Experiments were conducted in 3 replicates with similar results (2 *N. benthamiana* in each replicate treated at the same time). **B)** to **(C)** Co-IP assay shows AUR1 interacting with CCS52A2 and CCS52B. GFP-tagged AUR1 was co-expressed with Myc-tagged CCS52A2 **(B)** and CCS52B **(C)** in *N. benthamiana* leaves, the combination of GFP and CCS52-Myc were used as the negative control. The asterisk (*) indicates the target band. **D)** to **(E)** In vivo degradation assays indicate CCS52 promoting AUR1 degradation. HSC70 was the internal control (bottom). Representative immunoblot result and relative band intensities of AUR1-FLAG normalized to loading control protein were labeled. The value of co-expressing APC8 and AUR1 protein was set as 1.0. **F)** AUR1-FLAG protein levels are increased in *ccs52a2-1/+ ccs52b-1* mutant compared with Col-0. Anti-FLAG and Tubulin antibodies show the AUR1 protein and loading control (top 2). Target gene *AUR1* and house-keeping gene *ACTIN1* mRNA expression levels were analyzed (bottom 2 panels). Relative band intensities of AUR1-FLAG normalized to Tubulin were labeled according to 6 biological replicates (anthers isolated from 20 inflorescences of 3 independent lines of Col-0 and *ccs52a2-1/+ ccs52b-1* plants in each replicate are used). The value of samples with Col-0 background was set as 1.0. Source data are provided as a Supplementary data set S1 and supported by Supplementary Figs. S4 and S5A- to C.

In mitotic cell divisions, APC/C^Cdh1^ degrades Aurora kinase to facilitate exit from mitosis (Taguchi et al. 2002; Floyd et al. 2008). *Cdh1*, is an APC/C coactivator in mammals and *CCS52A* and *CCS52B* in plants (Tarayre et al. 2004). To test whether *CCS52A2* and *CCS52B* mediate the decay of AUR1, we examined their physical interaction with AUR1. Y2H assays demonstrate that CCS52A2/B interact with AUR1 (Supplementary Fig. S5D). Because full-length recombinant CCS52A2/B protein are poorly expressed in *E. coli*, so we constructed truncated forms including WD40 repeats (designated as CCS52A2_WD40_ and CCS52B_WD40_ in Supplementary Fig. S5E), which have been previously shown to contribute to protein–protein interaction (Schapira et al. 2017), pull-down assay confirmed their interaction (Supplementary Fig. S5F). We validated these results using the in vivo split-luciferase complementation assay, which shows strong and specific LUC activity with AUR1 and CCS52A2/B but not in negative controls (Fig. 5A). We confirmed these results using an independent Co-IP assay in *N. benthamiana* cells to show intact CCS52A2 and CCS52B are specifically co-immunoprecipitated with AUR1 but not the negative control (Fig. 5, B and C). Interestingly, the D-box of AUR1 is indispensable for interacting with CCS52A2/B, but dispensable for CDC20.1 in vivo (Fig. 5A). This demonstrates that the D-box of AUR1 is targeted by CCS52A2/B but not CDC20.1, suggesting that CCS52A2/B may be an upstream coactivator of AUR1. To test the effect of CCS52A2/B on APC/C-mediated AUR1 stability, we used the *N. benthamiana* leaf in vivo transient expression degradation assay and found that increasing amounts of CCS52A2/B have a notable effect on AUR1 stability (Fig. 5, D to E, Supplementary Fig. S5B and Supplementary data set S1).

Since both CDC20.1 and CCS52A2/B interact with AUR1, we examined how they influence the interaction of APC/C and AUR1. Using the split-luciferase complementation assay, we show that the addition of CDC20.1-GFP or GFP as a negative control do not diminish the interaction of AUR1 and APC10, but the interaction is significantly decreased by the addition of CCS52A2/B (Supplementary Fig. S5, G and I) (2-tailed Student's *t* test, ****P < 0.0001 in CCS52A2, ***P = 0.0007 in CCS52B). These results are consistent with immunoblotting data of protein extracts from the split-luciferase complementation assay tissues, which show that increasing CCS52A2/B is accompanied by declines of AUR1, while CDC20.1 or negative controls do not have decreased AUR1 (Supplementary Fig. S5H). These results suggest that coactivators CCS52A2/B assist APC/C-mediated ubiquitination and degradation of AUR1, while CDC20.1 has the opposite effect.

### Coactivators CCS52A2/B and CDC20.1 compete for APC/C binding in vivo

How do coactivators CCS52A2/B and CDC20.1 differentially affect their common interactor AUR1? APC/C coactivators have a conserved C-box and IR (isoleucine–arginine) tail that bind APC8 and APC3 subunits, respectively, to activate APC/C catalytic activity (Chang et al. 2015). Previous studies showed that coactivator CDC20 interacts with APC/C subunits (Xu et al. 2019; Lin et al. 2022), CCS52A2/B and CDC20.1 interact with full-length APC8 (Supplementary Fig. S6A) (Xu et al. 2019). We used the split-luciferase complementation assay to confirm these interactions in vivo (Supplementary Fig. S6B), supporting the idea that CCS52A2/B and CDC20.1 may act as APC/C coactivators. Given that coactivators have dual roles in determining substrate specificity and stimulation of APC/C catalytic activity, temporal regulation of coactivators is needed for orderly degradation of different substrates (Sivakumar and Gorbsky 2015). To investigate their relationship, we used a competitive split-luciferase complementation assay and found that addition of CCS52A2/B significantly diminished the association of APC8 and CDC20.1 (Supplementary Figure S6, B and D) (2-tailed Student's *t* test, *P = 0.0291, ***P = 0.0002), while the negative control does not (Supplementary Fig. S6, B to D), supporting the idea that coactivators CCS52A2/B and CDC20.1 may compete to interact with APC/C to regulate the degradation of distinct substrates, such as AUR1.

### CCS52A2/B are required for meiotic homologous alignment and segregation

CDC20 activity is required for meiosis in plants (Niu et al. 2015; Lin et al. 2022). *CCS52A2* and *CCS52B* are highly expressed in male meiocytes (Huang et al. 2019) (Supplementary Fig. S7A), suggesting a role in meiosis. To investigate whether *CCS52A2/B* are also required for meiosis, we obtained T-DNA insertional mutants of *ccs52a2* and *ccs52b* (Supplementary Fig. S7B), and validated them by RT-qPCR (Supplementary Fig. S7, C and D). The *ccs52b-1* mutant show normal vegetative growth, the *ccs52a2-2* mutant has pleiotropic phenotypes (Supplementary Fig. S7, E and F), consistent with previous report (Liu et al. 2012; Baloban et al. 2013). We also examined a second allele *ccs52a2-1*, and found it is homozygous lethal, we created a trans-heterozygote by crossing *ccs52a2-1/CCS52A2* as the male parent with *ccs52a2-2/ccs52a2-2* as the female parent. Col-0 plants produced siliques with an average of 51.3 viable seeds, while *ccs52a2-2* and *ccs52a2-1/ccs52a2-2* had an average of 13.5 and 13.7 seeds per silique, respectively (Supplementary Fig. S7J). Moreover, *ccs52a2-2* and *ccs52a2-1/ccs52a2-2* have reduced floral organs, fewer viable pollen grains per anther in *ccs52a2-2* (109 ± 38/anther, *n* = 27) (Student's *t* test, ****P < 0.0001) and in *ccs52a2-1/ccs52a2-2* (120 ± 40/anther, *n* = 19) (Student's *t* test, ****P < 0.0001) compared with Col-0 (463 ± 43/anther, *n* = 16) (Supplementary Fig. S7, G to H, K) and the residual pollen grains have aberrantly diverse sizes compared with Col-0. Male meiosis in Col-0 plants produces a uniform tetrad of microspores, but we observed atypical tetrads or polyads in *ccs52a2* mutants (Supplementary Fig. S7I), indicative of meiotic defect.

**Figure 6.**
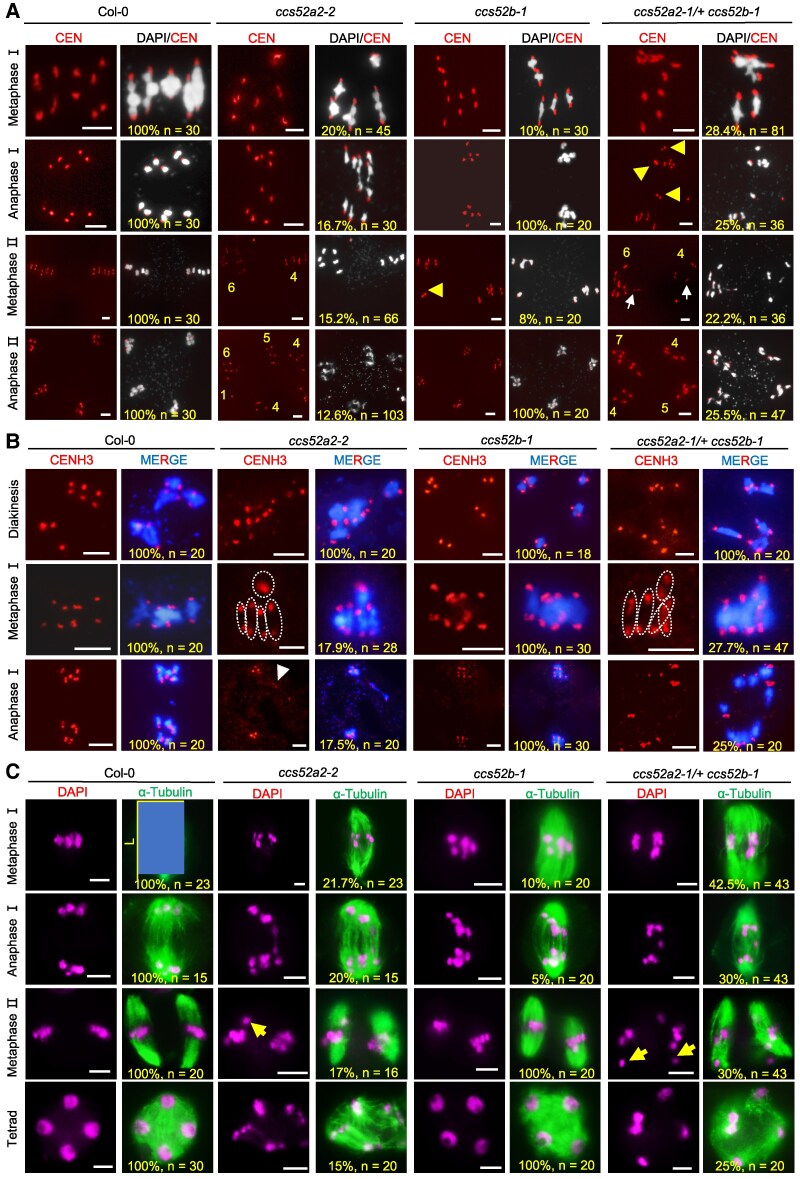
*CCS52A2* is required for the bivalent alignment and subsequent segregation. **A)** Meiotic chromosome behavior of Col-0, *ccs52a2-2*, *ccs52b-1*, and *ccs52a2-1/+ ccs52b-1* male meiocytes. White color indicates DAPI-stained chromosome and red color refers to centromere probe signal. Numbers indicate the chromosomes numbers segregated in male meiosis. The yellow arrowheads indicate misaligned centromere probe signal. The white arrows indicate the premature segregated sister chromatids. Scale bar, 5 *μ*m. **B)** Immunolocalization of CENH3/CENP-A in Col-0, *ccs52a2-2*, *ccs52b-1*, and *ccs52a2-1/+ ccs52b-1* male meiocytes. Anti-HTR12 (CENH3) antibody was used to indicate meiotic chromosomes. Blue color indicates DAPI-stained chromosome, red color refers to CENH3 signal. White dotted portions mark bivalents, and white arrowhead marks the bivalent with abnormal CENH3 signal and abnormal unsegregated bivalents. Scale bar, 5 *μ*m. **C)** Spindle morphology in Col-0, *ccs52a2-2*, *ccs52b-1*, and *ccs52a2-1/+ ccs52b-1* male meiocytes. The spindle was detected by immunostaining with antibody against Tubulin (green). Chromosomes were stained with DAPI. Yellow arrowhead indicates unusual chromosomes configuration. Horizontal and vertical yellow lines refer to the diameter (D) and length (L) of spindles, respectively. Scale bar, 5 *μ*m. The observed cell number and abnormal ratio were labeled in the Figures.

To investigate the meiotic defects more closely, we examined DAPI-stained chromosomes from male meiocytes and found that *ccs52a2* has no obvious defects in prophase Ⅰ, but at metaphase Ⅰ and Ⅱ, unlike the 5 well-aligned homologs in Col-0 and transgenic plants (*ProActin7:CCS52A2/ccs52a2-2*), the chromosomes in *ccs52a2* are misaligned on the equatorial plate, experience premature and uneven chromosome segregation, and form polyads or atypical tetrads (Supplementary Fig. S7L). The *ccs52a2* meiotic defects are rescued by trans-complementation with *CCS52A2* (Supplementary Fig. S7), supporting the idea that *CCS52A2* is required for male fertility and meiosis. Using fluorescence in situ hybridization (FISH) with a centromere probe, we found that, unlike the 5 pairs of centromere signals uniformly distributed at 2 poles observed in Col-0 meiocytes, 20% (*n* = 45) of *ccs52a2-2* meiocytes have asymmetrically aligned centromeres at metaphase I, 15.2% (*n* = 66) have abnormal cells at metaphase II with misaligned and unevenly distributed chromosomes and 12.6% (*n* = 103) develop into polyads or abnormal tetrads (Fig. 6A), suggesting that CCS52A2 is required for meiotic chromosome alignment and segregation. *ccs52b* mutant shows defects in chromosome alignment in metaphase, but subsequently forms normal tetrad.

Assembly and timely removal of cohesin are prerequisites for faithful chromosome segregation in meiosis. We analyzed the distribution of Arabidopsis meiosis-specific cohesin SYN1 at prophase I, and did not observe any difference between Col-0 and *ccs52a2-2* (Supplementary Fig. S8A), indicating that loading of SYN1 onto chromosomes is unaffected in *ccs52a2-2*, which is in general agreement with the previous studies of SYN1 localization in *apc8-1* and *cdc20.1-3* (Niu et al. 2015; Xu et al. 2019). Immunolocalization of the kinetochore marker protein CENH3 showed that 17.9% (*n* = 28) of *ccs52a2-2* meiocytes have asymmetric CENH3 signals at metaphase Ⅰ with one bivalent separated from others (Fig. 6B), and lagging bivalents on the equatorial plate at prophase II (Supplementary Fig. S8B), suggesting that CCS52A2 may participate in chromosome kinetochore orientation and correction of kinetochore-microtubule attachment errors.

During cell division, including meiosis, chromosome movement is facilitated by attachment of the kinetochores to spindle microtubules (Yao et al. 2000). We used immunostaining with antibodies against Tubulin to examine the spindle and observed that, 21.7% (*n* = 23) of *ccs52a2-2* meiocytes have twisted or narrow spindle morphology at metaphase Ⅰ and eventually formed multi-polar structures, consistent with chromosome mis-segregation (Fig. 6C). Furthermore, poor alignment of chromosomes is often related with extension of spindle poles at metaphase Ⅰ (Nagaoka et al. 2011; Kim et al. 2015), and *ccs52a2-2* have significantly longer spindles with an average length of 14.7 *μ*m (*n* = 23) compared with 11.5 *μ*m (*n* = 23) in Col-0 (Supplementary Fig. S8C) (Student's *t* test, ****P < 0.0001). These observations support the idea that chromosome misalignment and uneven or asynchronous segregation in *ccs52a2-2* meiocytes are likely related to aberrant kinetochore orientation.

Both CCS52A2 and CCS52B interact with AUR1, and are expressed during male meiosis. To test their potential redundancy, we generated double mutant plants. The double homozygous mutants are not viable so we analyzed plants that were heterozygous *ccs52a2-1/+* and homozygous *ccs52b-1/ccs52b-1*. Male meiocytes from *ccs52a2-1/+ ccs52b-1* have exacerbated phenotypic effects and showed a higher proportion of meiocytes with abnormal meiotic phenotypes, including asymmetrically aligned centromeres at metaphase Ⅰ (28.4%, *n* = 81) and metaphase Ⅱ (22.2%, *n* = 36), and development of polyads or abnormal tetrads (25.5%, *n* = 47) compared with the *ccs52a2-2* single mutant (Fig. 6A). Moreover, abnormal CENH3 distribution also increased to 27.7% (*n* = 47) in *ccs52a2-1/+ ccs52b-1* male meiocytes (Fig. 6B) and a higher ratio of *ccs52a2-1/+ ccs52b-1* (42.5%, *n* = 43) meiocytes have twisted or narrow spindle morphology at metaphase Ⅰ compared with *ccs52a2-2* (21.7%, *n* = 23) (Fig. 6C). Taken together, these results suggest that *CCS52A2* and *CCS52B* are partially functional redundancy in meiotic chromosome alignment and segregation.

### AUR1 levels decrease in *cdc20.1* and increase in *ccs52* mutants

To investigate the meiotic defects in *ccs52*, we transformed AUR1-FLAG into *ccs52a2-1/+ ccs52b-1* heterozygous mutants, used anthers of stable AUR1-FLAG transgenic Arabidopsis plants to examine AUR1 protein levels and found significantly higher AUR1 levels (Student's *t* test, ****P < 0.0001) in *ccs52a2-1/+ ccs52b-1* relative to Col-0 (Fig. 5F and Supplementary Fig. S5A). Attempts to obtain a native Aurora antibody were not successful, so as an alternative we validated AUR1 levels by examining histone H3 phospho-Ser10 (H3S10) levels, which has previously been used as a marker of Aurora activity and is highly conserved in mammals, yeast and plants (Ditchfield et al. 2003; Kawabe et al. 2005; Niu et al. 2015). We used immunofluorescence assays with antibodies against H3S10ph together with a component of the synaptonemal complex (SC)-transverse filament ZYP1 for marking pachytene chromosomes. We classified H3S10ph localization into 3 patterns based on phenotypic distribution (type I, II, and III), and observed a significantly higher proportion of patch and continuous H3S10ph signal along the chromosome (type III pattern) in *ccs52* mutant chromosome spreads (Fisher's exact test, ****P < 0.0001, type III) (Supplementary Fig. S8, D and E). Compared with Col-0, H3S10ph fluorescence signal was weaker in *cdc20.1-3* (*n* = 56) (Fisher's exact test, *P* = 0.06, type I) (Supplementary Fig. S8D) and *cdc20.1-3 AUR^RNAi^* (*n* = 66) (Fisher's exact test, **P = 0.0056, type I) (Supplementary Figs. S8E and S9B), but increased in *ccs52* mutant, providing additional evidence to support altered AUR1 levels in corresponding mutant backgrounds. Taken together, these results suggest that APC/C and its coactivators are required for maintaining the AUR1 homeostasis.

### APC8, CCS52, and CDC20.1 act in the same pathway during meiosis

APC/C and its coactivators are both required for alignment and segregation of homologous chromosomes during meiosis. Meiosis-specific *AUR1* knockdown (*AUR^RNAi^*) (Niu et al. 2015) has defects in meiotic chromosome alignment and segregation similar to *apc8-1*, *cdc20.1*, *ccs52a2-1/+ ccs52b-1* mutants. To test their genetic relationship, we crossed *apc8-1*, *cdc20.1-3*, and *ccs52a2-1/+ ccs52b-1* with *AUR^RNAi^* to obtain high-order mutants. RT-qPCR analysis showed significant attenuation of *AUR1* expression in *apc8-1 AUR^RNAi^* (Student's *t* test, **P = 0.0029)*, cdc20.1-3 AUR^RNAi^* (Student's *t* test, **P = 0.0038) and *ccs52 AUR^RNAi^* (Student's *t* test, *P = 0.0387) mutants compared with each of the single mutants (Supplementary Fig. S9, A to C). Analysis of chromosome morphology by FISH with a centromere probe showed that male meiocytes from these higher-order mutants displayed meiotic chromosome defects including asynchronously alignment at the equatorial plate and uneven segregation at anaphase, resembling each of the single mutants (Supplementary Fig. S9D). We also crossed *apc8-1* with *ccs52a2-1/+ ccs52b-1*. Unfortunately, the triple mutants have an extreme dwarfing phenotype and cannot be used for analyzing meiotic phenotypes (Supplementary Fig. S9E). Taken together, these data support the idea that APC8, coactivators CDC20.1/CCS52 and AUR1 function in the same genetic pathway during meiosis.

## Discussion

### Identification of AUR1 as a substrate for APC/C during Arabidopsis meiosis

APC/C is a multi-subunit Cullin-RING ubiquitin ligase that ensures an orderly cell cycle, including meiosis (Peter et al. 2001; Xu et al. 2019). Recent studies revealed meiotic targets of APC/C (Willems and De Veylder 2022) include Cyclin B1 and the cohesin protectors Securin and Shugoshin1 in mouse oocytes (Herbert et al. 2003; Rattani et al. 2017), yeast (Buonomo et al. 2000) and *C. elegans* (Siomos et al. 2001), and Shugoshin1, MPS1 and Cdh1 in yeast (Jonak et al. 2017). Less is known about the homologs of these proteins in plants and the direct meiotic targets of APC/C in plants have not been defined. PANS1/RSS1 (Cromer et al. 2019) and SWITCH1 (Yang et al. 2019), homologs of cohesin protectors Securin and Sororin (Rankin et al. 2005), respectively, may be APC/C substrates in plants, but direct biochemical evidence is still lacking. Aurora kinases, which are critical component of the SAC, play multiple roles in assisting faithful chromosome segregation from yeast to humans during both meiosis and mitosis including mediating correction of erroneous kinetochore-microtubule attachment, kinetochore orientation, and spindle microtubule assembly (Hauf et al. 2007; Yoshida et al. 2015; Shrestha et al. 2017; Berthezene et al. 2020; Wellard et al. 2020). Here, we present several pieces of evidence that show AUR1 is a meiotic APC/C substrate in plants. We previously showed that *cdc20.1-3* mutant phenocopies the meiotic defects in plants with meiosis-specific knockdown of *AUR1* and *apc8-1* mutants (Niu et al. 2015; Xu et al. 2019), supporting the idea that *APC/C*, *CDC20.1* and *AUR1* are functionally related in SAC-dependent meiotic chromosome segregation. We used ubiquitin-modified proteome analysis combined with genetic and biochemical approaches to show that the AUR1 is required for *CDC20.1*'s function in meiosis (Fig. 1 and Supplementary Fig. S1). In addition, our biochemical experiments provide strong evidence that APC/C mediates AUR1's ubiquitination and degradation (Fig. 2 and Supplementary Figs. S3 and S4). Taken together, this study shows that AUR1 is a meiotic substrate of APC/C in Arabidopsis meiosis.

### Molecular mechanism of APC/C-mediated meiotic chromosome segregation

Coactivators regulate the catalytic activity and substrate specificity of APC/C (da Fonseca et al. 2011). We showed coactivators *CCS52A2*/*B*, the homologs of *Cdh1/Fzr1* in yeast, but not *CDC20.1*, regulate AUR1 protein stability in meiosis (Figs. 4 and 5 and Supplementary Fig. S5). Aurora protein homeostasis is maintained by APC/C^CCS52A2/B^-mediated ubiquitination and subsequent degradation to facilitate SAC-dependent meiotic chromosome segregation. Considering the highly conserved structure and function of the APC/C E3 ubiquitin ligase in multiple species, as well as the fact that ubiquitination and degradation are shared mechanisms in both mitosis and meiosis, the degradation of Aurora kinase by APC/C may be a general mechanism in eukaryotic cell cycle control. This conjecture is supported by previous reports in human cells and *Xenopus* eggs that APC/C^Cdh1^ recognizes the KEN motif, D-box or A-box of Aurora kinases for proteometabolism at the end of cell division (Castro et al. 2002; Taguchi et al. 2002; Nguyen et al. 2005; Floyd et al. 2008). Unlike the proteometabolism that occurs after chromosome segregation, we suggest that Aurora's degradation and homeostasis help facilitate SAC-dependent homologous and sister chromosome alignment and segregation in Arabidopsis male meiocytes.

The role of coactivator Cdh1 homologs CCS52 in plants during meiosis remains unclear. Although the double *CCS52a and CCS52b* mutants are homozygous lethal, we obtained *ccs52a2/+ ccs52b-1* mutants and found that *CCS52A2/B* have a role in meiotic chromosome alignment and segregation by controlling SAC-dependent spindle assembly and correction of erroneous kinetochore-microtubule attachment in Arabidopsis male meiocytes (Fig. 6 and Supplementary Fig. S7). Consistent with this, immunofluorescence results using antibody against Aurora marker H3S10ph and immunoblotting for protein levels demonstrates that AUR1 protein degradation is impeded in *ccs52a2-1/+ ccs52b-1* compared with Col-0 (Supplementary Fig. S8, D and E; Fig. 5F), which may be an aspect of the meiotic defects in *ccs52* mutants. Furthermore, inappropriate amounts of Aurora kinases resulting in meiotic SAC dysfunction with increased aneuploidy and decreased fertility were also found in several species from yeast to humans (Ma et al. 2003; Nguyen et al. 2005; Swain et al. 2008; Jordan et al. 2009; Demidov et al. 2014; Niu et al. 2015; Aboelenain and Schindler 2021; Nikalayevich et al. 2022). Our genetic evidence using stable transgenic plants overexpressing AUR1K102A, which experiences less ubiquitination, demonstrates that the inappropriate accumulation of Aurora causes meiotic abnormalities and aneuploidy (Supplementary Fig. S2), which is consistent with previous finding in Arabidopsis (Demidov et al. 2014). Similarly, overexpression or repression of SAC components BUB1, BubR1 and Mad2 in mouse (Jeganathan et al. 2007; Ricke et al. 2011), AURB and Mad1 in human cells (Nguyen et al. 2005; Zhao et al. 2021) also lead to chromosome alignment defects and aneuploid progeny. Therefore, maintaining optimal protein levels of SAC components may be critical for their normal function in ensuring an orderly cell cycle, including meiosis. Unlike the role of APC/C in controlling chromosome segregation through regulation of the timely removal of cohesin from yeast to mammals (Buonomo et al. 2000; Herbert et al. 2003; Reis et al. 2007; Jonak et al. 2017), we found that APC/C modulates the ubiquitination and degradation of AUR1 in SAC-dependent meiotic chromosome segregation in Arabidopsis male meiocytes.

### Coactivators coordinate to fine tune AUR1 homeostasis to sustain meiotic SAC function

Coactivators have been reported to coordinate sequential control of APC/C catalytic activity in mammals (Fülöp et al. 2005; Homer et al. 2009). In mouse oocytes, coactivator Cdh1 dominates meiotic prometaphase Ⅰ progression, and subsequently coactivator CDC20 takes part in cell cycle control (Homer et al. 2009). Both Cdh1 and CDC20 synergistically influence meiotic nuclear division and meiotic exit in yeast (Chikashige et al. 2017; Ostapenko and Solomon 2024). However, whether coactivators have similar functions in plant meiosis has been unclear. Here, we demonstrate that Arabidopsis APC/C coactivators CCS52A2/B compete with CDC20.1 (Supplementary Figure S6), which is consistent with previous reports that Cdh1 may impede the interaction between CDC20 and APC/C in human sperm cells (Tanno et al. 2020) and modulates the timing of APC/C^CDC20^ activity (Holt et al. 2012; Rattani et al. 2017). In addition, the meiotic phenotype of *ccs52* mutants (Fig. 6) and *cdc20.1* mutant reported previously (Niu et al. 2015) both suggest that they act early in meiosis to regulating SAC function. In addition, previous studies in mouse germ cell meiosis showed that CCS52 homologous protein CDH1/FZR1 regulates APC/C activity by controlling its association and dissociation from APC/C through dephosphorylation and phosphorylation (Tanno et al. 2020). A similar mechanism may be employed here. CCS52 and CDC20.1 competing for APC/C might be regulated by phosphorylation or protein–protein interaction. To establish a mechanistic link between AUR1, CDC20.1, and CCS52A2/B in the regulation of Arabidopsis male meiosis, the generation of native antibodies for cytological localization studies in meiocytes and comprehensive meiotic phenotypic characterization of available higher-order mutants (*aur*, *cdc20.1*, and *ccs52a2*/*b*) would conclusively demonstrate their relationship in mediating faithful meiotic chromosome segregation in the future.

**Figure 7.**
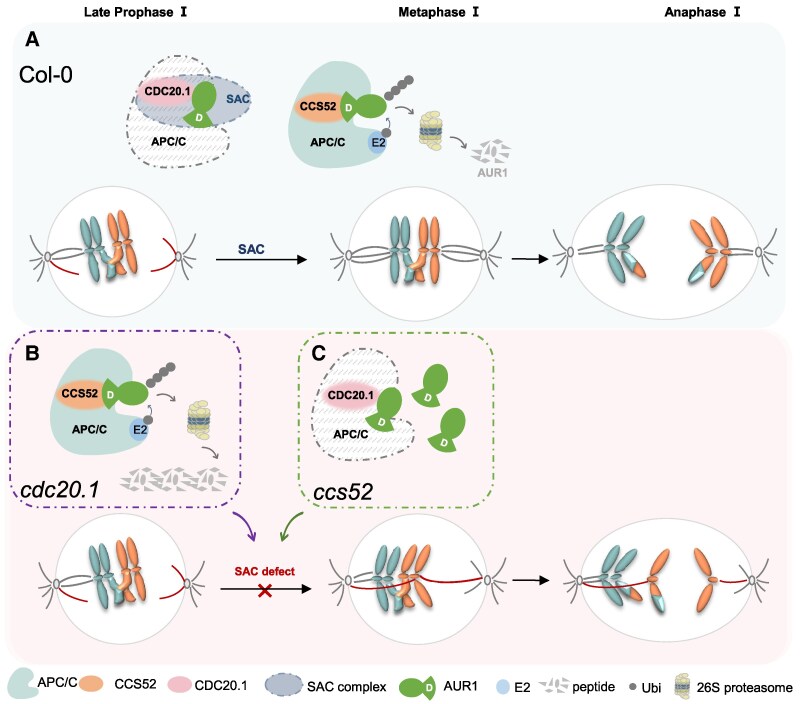
A model showing APC/C^CCS52^-mediated ubiquitination and degradation of AUR1 for meiotic chromosome alignment and segregation. In Col-0 **(A)**, at late prophase Ⅰ, CDC20.1 interacts with AUR1 as SAC component to limit APC/C^CDC20.1^ catalytic activity. CDC20.1 simultaneously competes with CCS52 to binding APC/C, thus inhibiting APC/C^CCS52^-dependent premature ubiquitination and degradation of AUR1. SAC surveillance mechanism perceives and monitors unsatisfied kinetochore-microtubule attachment or abnormal spindle assembly, AUR1 kinase accomplishes erroneous correction to ensure bivalents align well on the equatorial plate at metaphase and homologs segregated faithfully. In contrast, in *cdc20.1*  **(B)**, CCS52 functions as APC/C coactivator to prematurely degrade AUR1 due to lack of coactivator CDC20.1 competitional inhibition, which causes failure in correcting erroneous kinetochore-microtubule attachment as well as the spindle assembly, thus leading to the misalignment and abnormal chromosome segregation afterward. Similarly, in *ccs52*  **(C)**, CDC20.1 acts as coactivator of APC/C but does not degrade AUR1, which results in improper accumulation of AUR1, thus causing SAC dysfunction and ultimately fails in faithful chromosome segregation in meiosis.

Based on our results and previous reports, we propose a working model illustrating how APC/C and its coactivators coordinately regulate SAC function through targeting AUR1 to ensure faithful chromosome segregation in Arabidopsis meiosis (Fig. 7). At late prophase Ⅰ, CDC20.1 interacts with SAC component AUR1 (Fig. 4). Similar to previous reports in early mitosis that CDC20.1 plays a role in SAC function through physical interaction with SAC effectors to prevent anaphase onset in Arabidopsis (Kevei et al. 2011; Niu et al. 2015; Deng et al. 2024b), here CDC20.1 may act as an SAC component and prevent the activation of APC/C for ubiquitinating substrates such as AUR1, thereby ensuring normal SAC function (Fig. 4). A recent report also supports the idea that Slp1 (homolog of CDC20) phosphorylation-mediated instability acts in concert with SAC activation in fission yeast (Sun et al. 2022). CDC20.1 competes with CCS52 for APC/C interaction (Supplementary Fig. S6), imposing dynamic regulation of APC/C dependent ubiquitination and degradation of SAC component including AUR1, thereby ensuring normal SAC function. The SAC surveillance mechanism perceives and monitors erroneous kinetochore-microtubule attachment or abnormal spindle assembly (Musacchio and Salmon 2007). As an SAC component, AUR1 kinase helps facilitate error correction. CCS52 competes with CDC20.1 for APC/C activation (Supplementary Fig. S6) and AUR1 is degraded by APC/C^CCS52^, which ensures proper alignment of bivalents on the equatorial plate and their subsequent accurate segregation (Niu et al. 2015). In contrast, in the absence of *CDC20.1*, the interaction of CCS52 with APC/C is not appropriately regulated, which leads to premature or overactive degradation of AUR1, causing defects in SAC function and a failure to correct erroneous kinetochore-microtubule attachment or unusual spindle assembly, resulting in misalignment of chromosomes and abnormal segregation. Similarly, in the absence of *CCS52*, AUR1 is not appropriately degraded and accumulates (Fig. 5F and Supplementary Fig. S8, D and E) because of failure to activate APC/C. Subsequently, improper spindle configurations develop, chromosomes become misaligned and meiotic segregation errors occur. In summary, our results reveal mechanistic insights into APC/C function and the antagonism among APC/C coactivators in regulating AUR1 homeostasis during plant meiosis.

## Materials and methods

### Plant materials and growth conditions

All T-DNA insertional mutants and transgenic plants were in the *Arabidopsis thaliana* Columbia-0 (Col-0) ecotype background. The T-DNA insertional mutant *cdc20.1-3*, and *ProDMC1:Aurora1^RNAi^* (Niu et al. 2015), the *apc8-1* mutant and the *APC8-YFP* transgenic complementation plants were reported previously (Zheng et al. 2011; Xu et al. 2019). *ccs52a2-1* (SALK_001978), *ccs52a2-2* (SALK_073708), *ccs52b-1* (SALK_098269) were obtained from the Arabidopsis Biological Resource Center (ABRC), the homozygous lines were identified by PCR, *ccs52a2-2* (SALK_073708) homozygous mutants were used for FISH and immunostaining. The high-order mutants were generated by crossing corresponding mutants mentioned above. Primers used for genotyping were listed in Supplementary Table S2. Seeds were plated on half-strength Murashige and Skoog medium (½MS) medium after sterilizing with 75% ethanol, and seedlings were transplanted onto soil and grown in a greenhouse with a photoperiod of 16 h day/8 h night at 22 ℃ with 70% humidity. *N. benthamiana* was grown in the same conditions as Arabidopsis.

### Bacterial strains and cultural condition

*E. coli* BL21 (DE3) was used for expressing proteins. Bacteria were cultured overnight at 37 ℃ on Luria-Bertani (LB) solid medium supplemented with antibiotics, including kanamycin (50 *μ*g/mL) and ampicillin (50 *μ*g/mL). Cultures were used to inoculate fresh broth media with antibiotics and incubated for another 16 h at 18 ℃. The *Agrobacterium tumefaciens* strain GV3101 was used to generate transgenic Arabidopsis and for transient expression in *N. benthamiana* cells. *A. tumefaciens* carrying recombinant plasmids were cultured at 28 ℃ for 2 d on LB solid medium with kanamycin, gentamycin (50 *μ*g/mL) and rifampicin (25 *μ*g/mL) then used to incubate fresh broth medium for at least 12 h when OD_600_ reached to 2.0. Yeast Y187, Y2H gold, and AH109 strains were incubated at 28 ℃ for 2 to 3 d for yeast Y2H experiment.

### Recombinant plasmids and plant transformation

Full-length coding sequences (CDS) of *AUR1*, *CCS52A2*, and *APC8* from Col-0 cDNA were amplified and cloned into a modified pCAMBIA1306 plasmid (digested by BamHⅠ/SalⅠ) driven by the *Actin7* promotor with the FLAG epitope tag at the C terminus using the One-step Cloning Kit (Novoprotein, China) or T4 ligase according to the manufacturer protocols for *A. tumefaciens*-mediated transformation. Plant expression vector pCAMBIA1306 (1×35S Promotor) with the FLAG, GFP or Myc epitope tag at the C terminus after BamHⅠ or XbaⅠ and SalⅠ digestion were ligated with cloned *AUR1*, *CCS52A2*, *CCS52B*, *CDC20.1* cDNA for biochemical assays. D-box truncated AUR1 (△D-box) and ubiquitination site mutant (K102A) were cloned using the full-length AUR1 as the template. The position of AUR1 truncations and point mutation are indicated in Supplementary Fig. S1C. Primers used in this study were shown in Supplementary Table S2, and all constructs were verified using Sanger sequencing.

Transgenic lines were obtained through introducing *ProActin7:AUR1-FLAG*, *ProHTR2:AUR1-FLAG, ProDMC1:AUR1-FLAG, ProCDC20.1:AUR1-FLAG, ProHTR2:AUR1K102A-FLAG, ProDMC1:AUR1K102A-FLAG, ProCDC20.1:AUR1K102A-FLAG, ProActin7:CCS52A2-FLAG*, *ProActin7:APC8-FLAG* vectors *A. tumefaciens* stain into Col-0 or corresponding mutants by the floral-dip method (Clough and Bent 1998). The T_0_ and T_1_ plants were screened in ½MS medium with 25 mg/L hygromycin. Plasmid harboring AUR1-FLAG was transferred into *cdc20.1-3* heterozygous mutant to segregate stable inherited transgenic plants in Col-0 and *cdc20.1-3* homozygous plants, which were crossed with *apc8-1*, *APC8-YFP* overexpressing plant to analyze the AUR1 protein level. The same construct above was also introduced into *ccs52a2-1 ccs52b-1* heterozygous mutants, anthers of AUR1-FLAG transgenic Arabidopsis plants to examine AUR1 protein levels.

The CDS of *AUR1*, *AUR1*△*D-box* were cloned into a modified His fusion epitope expression vector *pET28a* and *pET28a-SUMO* vectors (digested with BamHⅠ and EcoRⅠ or SalⅠ), the full-length CDS or truncated forms of *APC10*, *CDC20.1*, *CCS52A2, and CCS52B* were cloned into a modified GST fusion protein expression vector *pGEX-4T-1* (digested by BamHⅠ and SalⅠ or XhoⅠ) for pull-down or other biochemical assays.

Full-length CDS sequence of *AUR1* and various truncated *AUR1* were amplified using full-length cDNA of *AUR1* as template, *CDC20.1*, *CCS52A2*, *CCS52B* were cloned into *pGBKT7* vector (digested by NdeⅠ or EcoRⅠ/ EcoRⅠ or SalⅠ or BamHⅠ) (BD, Clontech); full-length CDS sequences of *APC10*, *APC8*, *CDC20.1*, *CCS52A2*, *CCS52B* were cloned into *pGADT7* vector (digested by NdeⅠ and BamHⅠ) (AD, Clontech) for yeast Y2H; Full-length CDS sequences of *AUR1*, *AUR1*△*D-box*, *CDC20.1* were amplified and cloned into *JW771* (*nLUC*) vector (digested by BamHⅠ and SalⅠ), corresponding CDS sequences of *CDC20.1*, *CCS52A2*, *CCS52B*, *APC10*, *APC8* were amplified and cloned into *JW772* (*cLUC* vector) (digested by KpnⅠ and SalⅠ) for split-luciferase complementation assay.

### Morphological plants and chromosomes analyses

Plants were photographed with an iPhone rear-facing camera. Inflorescences containing 4 to 7 stage anther were fixed in a modified Carnoy's fixative (3 ethanol:1 acetic acid) for >2 h in ice water. Alexander red staining of pollen grains was performed by dyeing anthers at 65 ℃ for 0.5 h (Peterson et al. 2010). Tetrads, centromere FISH, chromosome spreading and immunofluorescence assay were performed as previously described (Wang et al. 2014).

Rabbit Anti-SYN1 polyclonal antibody (Shanghai AnGo Biotechnology CO), Rabbit Anti-ZYP1 polyclonal antibody (Shanghai AnGo Biotechnology CO), Rabbit Anti-CENH3 polyclonal antibody (ab72001, Abcam), Rabbit Anti-H3S10ph polyclonal antibody (CAT#GTX128116, GeneTex) and Mouse anti-Tubulin antibody (M20005, Abmart) were diluted 1:200 to detect corresponding immunofluorescence signal as previously used (Chelysheva et al. 2010; Wang et al. 2014). Secondary antibody Alexa Fluor 555 Goat Anti-Rabbit IgG/488 Goat Anti-mouse IgG (H + L) (A-21428) (Invitrogen, USA) were diluted 1:1000. Both images were recorded using a Zeiss Axio imager A1 microscope (Zeiss, German). Alexa Fluor 555: Filter set 31 Cy 3.5 shift free (E) Excitation Bandpass (EX BP) 550 to 580 nm, Beam Splitter Filter (BS FT) 585 nm, Emission Bandpass (EM BP) 590 to 650 nm, × 100 objective 5.5 mW; Alexa Fluor 488: Filter set 46 YFP shift free (E) EX BP 490 to 510 nm, BS FT 515 nm, EM BP 520 to 550 nm, × 100 objective 1.6 mW;DAPI: Filter set 49 DAPI shift free (E) EX G 365 nm, BS FT 395 nm, EM LP 420 to 470 nm, × 100 objective 5.5 mW.

### RNA extraction and RT-qPCR

The 4 to 7 stages inflorescences of Col-0 and mutants were used to extract mRNA using Trizol reagent (Invitrogen) then cDNA was synthesized using a PrimeScript RT reagent kit with DNA Eraser (Cat. no. RR047A; TaKaRa). Reverse transcription products were used as the template for RT-qPCR using step one plus real-time PCR system (Applied Biosystems, Carlsbad, CA, USA) with iTaq Universal SYBR Green Supermix (Cat. no. 72-5124; Bio-Rad) with Bio-Rad CFX96 Touch Thermocycler (Bio-Rad) and data were collected and analyzed with Bio-Rad CFX Manager (v3.1). After the normalization of cycle threshold (CT) value to*ACTIN2*, the relative expression was calculated according to 2^−ΔΔCt^ (△Ct (C_t, gene of interest_ − C_t, actin2_) and △△Ct (△C_t_ −△C_t, control_)) method (Schmittgen and Livak 2008; Bustin 2010). *ACTIN1* and *ACTIN2* were internal control. Three biological replicates and 3 technical replicates for each biological replicate and each transgenic plants includes at least 2 independent lines were performed and the final statistical significance of gene expression levels was using the 2-tailed Student's *t* test.

### Yeast Y2H

Full-length CDS sequence of *AUR1* and various truncated *AUR1* were amplified using full-length cDNA of *AUR1* as template, *CDC20.1*, *CCS52A2*, *CCS52B* were cloned into *pGBKT7* vector (digested by NdeⅠ or EcoRⅠ/ EcoRⅠ or SalⅠ or BamHⅠ) (BD, Clontech); Full-length CDS sequences of *APC10*, *APC8*, *CDC20.1*, *CCS52A2*, *CCS52B* were cloned into *pGADT7* vector (by NdeⅠ and BamHⅠ) (AD, Clontech) for yeast Y2H. Corresponding recombinant vectors transformed into Y187 yeast strain and Y2H gold yeast strain respectively, or *pGADT7* and *pGBKT7* recombinant vectors co-transformed into AH109 stain. After mating/growing on YPDA medium for 48 h, transformants were selected in the synthetic dropout media (SD) without Trp, Leu, His, and Ala (SD/-WLHA) plates and SD/ -Trp-Leu-His (SD/-WLH) and SD/- Trp-Leu (SD/-WL) for several days.

### Pull-down assay

All these constructs were transformed into *E. coli* BL21 (DE3) or Rosetta (DE3) strain and promoted protein expression at 18 ℃ for 18 h with the addition of 0.2 mm Isopropyl β-D-1-thiogalactopytanoside (IPTG). Recombinant proteins were purified with Glutathione Sepharose beads (Merck, Germany) and Ni-NTA His-bind Resin (Merck, Germany) according to the manufacture protocols.

For each group, 4 *μ*g purified proteins with different epitopes were incubated in 500 *µ*L pull-down binding buffer (300 mm NaCl, 50 mm Tris-HCl pH 8.0, 1 mm PMSF, 1 mm DTT) on a rotating wheel at 4 ℃ for 4 h, then incubating with Ni-NTA beads for 2 h, washing the protein-bound beads with washing buffer (150 mm NaCl, 50 mm Tris-HCl pH 8.0, 1 mm PMSF) for 8 times and removed unbound proteins then the final beads were boiled in SDS loading buffer, the pull-down proteins were analyzed by immunoblot with anti-GST (1:2000, Abmart, China), His antibody (1:2000, Abmart, China), and anti-SUMO antiserum (1:1000, Abcam).

### Transient expression in *N. benthamiana* leaves, protein extraction and IP assay

Plant expression vector pCAMBIA1306 (1×35S Promotor) with the FLAG, GFP or Myc epitope tag at the C terminus after BamHⅠ or XbaⅠ and SalⅠ digestion were ligated with cloned *AUR1*, *CCS52A2*, *CCS52B*, *CDC20.1* cDNA introduced into *A. tumefaciens* stain GV3101 (Weidi Biotechnology, China). *A. tumefaciens* strains were cultured at 28 ℃ for 2 d and used to inoculate liquid LB medium with corresponding antibiotics and 40 μμ acetosyringone (AS) to induce expression of corresponding target proteins in an incubator shaker at 28 ℃ for 16 h. When the concentration reached OD_600_ = 2.0, the bacteria were collected by gentle centrifugation at 5,000 g for 10 min. The precipitated bacteria were resuspended in buffer (10 mm MgCl_2_, 200 *μ*M AS) to a final OD_600_ = 1.0 and allowed to rest for 1 to 3 h at room temperature before injection into the bottom surface of 4-wk-old *N. benthamiana* leaves (Liu et al. 2010). Crude proteins were extracted by grinding in liquid nitrogen with native lysis buffer (50 mm Tris-HCl pH 8.0, 150 mm NaCl, 10 mm MgCl_2_, 3‰ NP40, 5% glycerol) supplemented with 5 mm DTT (dithiothreitol), 1 mm PMSF, protease inhibitor 100× cocktail (YEASEN, China). Extracts were centrifuged at 12,000*g* at 4 ℃ for 30 min, and the supernatants was harvested.

For IP or Co-IP assays, protein supernatants were incubated with corresponding affinity beads (anti-FLAG agarose beads magnetic beads (Sigma, M8823, USA), anti-GFP magnetic agarose beads (Merck, 70541, Germany), TUBE2 affinity gel matrix (LifeSensors, UM402, USA) at 4 ℃ for 4 h, followed by washing 3 times to remove unbound protein, the bead-bound proteins were denatured for western blot analysis, anti-GFP (1:2000, GNI, Japan), anti-FLAG (1:2000, GNI, Japan) and anti-Myc (1:2000, Sigma, Germany), anti-UBQ11 (1:10000, Agrisera, Sweden), anti-Tubulin antibody (1:2000, Abmart, China), anti-HSC70 (1:2000, ENZO, USA) goat anti-rabbit/mouse IgG HRP-conjugated (1:2000, GNI, Japan) antibodies were used to detect corresponding proteins. Proteins blots were imaged with a Clinx-3400 chemiluminescence imaging system.

### Split-luciferase complementation assay

This assay was performed as described previously (Chen et al. 2008). Briefly, full-length CDS of *AUR1*, *AUR1*△*D-box*, *APC10*, *APC8*, *CDC20.1*, *CCS52A2*, *and CCS52B* were fused in frame with the C-terminal fragment and N-terminal fragments of luciferase (cLUC or nLUC). GUS-nLUC and GUS-cLUC were used as negative controls. Different combinations were transformed into *A. tumefacien* strain GV3101 and infiltrated into *N. benthamiana* leaves along with P19. For competitive split-luciferase complementation assay, CDC20.1, CCS52A2, and CCS52B or mock (empty vector with Myc tag) were added to this system to detect their effect on the interaction as previously described (Zhong et al. 2019). After processing with luciferin, Luciferase luminescence of the infiltrated leaves was detected by a NightShade LB 985 system (Berthold Technology, Germany). Data analysis was performed with indiGo software (v2.0.5.0).

### In vivo and semi-in vivo protein degradation assay

For in vivo protein degradation assay using *N. benthamiana*, diverse combinations of *A. tumefaciens* carrying various constructs were co-infiltrated and transiently expressed for 2 d. The sample were extracted and detected by immunoblot then detected the transcriptional expression level as described previously (Liu et al. 2010).

For in vivo protein degradation assay in Arabidopsis, inflorescences of *ProActin7:AUR1-FLAG/Col-0* and *ProActin7:AUR1-FLAG/apc8-1* were collected and proteins were extracted with native extraction buffer containing CHX, with or without 50 *μ*M MG132 (Sigma-Aldrich, USA), and then were examined by immunoblot with anti-FLAG, anti-HSC70 antibody after reacting for indicated time courses at room temperature.

For the *semi-*in vivo protein degradation assay in *N. benthamiana* cells, APC8 and AUR1 proteins were transiently expressed and extracted in a native lysis buffer. The equivalent mixture of AUR1-FLAG extracts and APC8-Myc or mock extracts with addition of a final concentration of 10 *μ*M ATP were incubated at room temperature for the indicated time points. Control groups containing MG132 were set up under the same conditions and specific proteins in the extracts were detected by immunoblotting.

### Cell-free degradation assay

A cell-free degradation assay was performed as previously described (Garcia-Cano et al. 2014). Total protein from Col-0 and *apc8-1* inflorescences were extracted in native lysis buffer with addition of 10 mm ATP and adjusted to equal concentrations. Purified recombinant SUMO-AUR1 and SUMO-△D-box were mixed with protein lysates and incubated for the indicated time periods at room temperature in the presence or absence of 50 *μ*M MG132. SUMO-tagged proteins were detected using an anti-SUMO antiserum. Three independent experiments were carried out and Image J software was used for quantitative analysis. A cell-free decay assay was performed as previously described (Ruan et al. 2019). Equal amounts of SUMO-AUR1 were incubated with native extracts from Col-0 and increasing amounts of CDC20.1 protein. IP products of inflorescence from transgenic plant expressing CDC20.1-FLAG and purified recombinant GST-CDC20.1 protein were used. Reaction products were detected by immunoblot.

### In vitro ubiquitination assay

Equal amounts of His-tagged recombinant AUR1 proteins immobilized on Ni-NTA affinity agarose beads were incubated with equal amounts of Col-0, *apc8-1* and *APC8-YFP* protein lysates at 25 ℃ for 6 h in native protein extraction buffer with 50 *μ*M MG132 as previously described (Wang et al. 2013). Beads bound by His-AUR1 were separated from protein supernatants and boiled with SDS-PAGE loading buffer after washing 5 times. Then samples were analyzed by immunoblots using anti-His and anti-UBQ11 antibody.

### Quantification and statistical analysis

GO analysis was performed according to agriGO website (http://bioinfo.cau.edu.cn/agriGO/index.php) and statistics were calculated by Fisher's exact test, multi-test adjustment method (Yekutieli-FDR under dependency). The overlaid dot plots in bar graphs were prepared using GraphPad Prism 7.00 software or Excel 2019 (Microsoft) for calculating the mean and standard deviation (SD) of pollen numbers and the remaining protein levels. Statistical analyses were performed using 2-sided 2-tailed Student's *t* test, 2-tailed Fisher's exact test, significance was determined on the basis of 95% confidence intervals (*P < 0.05; **P < 0.01; ***P < 0.001; ****P < 0.0001; ns, no significant, *P* ≥ 0.05). Quantification analysis was shown as mean ± SD. Adobe Photoshop 2023 software was used for resizing and adjusting the images. ImageJ software was used to quantify western blot bands, relative band intensities normalized to control (HSC70 or Tubulin) were labeled above the pictures. Biological replicate numbers are indicated in figure legends. The relative fluorescence intensity of luciferase in *N. benthamiana* leaves was calculated and normalized to negative control group using indiGo software. Each batch of *N. benthamiana* was used as a biological replicate to exclude individual difference in *N. benthamiana*, which ensures good reproducibility of the experiments. The level of negative control in each independent experiment was set as 1.0, while the experimental groups were calculated based on the control group in each independent experiment, a relative value compared with the control, then the final statistical data were plotted based on the relative value in experimental groups and “1.0” in control group in biological replicates. Detailed statistical data with 2-tailed Student's *t* test and Fisher's exact test for all relevant figures were provided in Supplementary data set S1.

### Accession numbers

The genes discussed in this paper can be found in the Arabidopsis Genome Initiative database TAIR (https://www.arabidopsis.org/) as follows: *AUR1* (AT4G32830), *APC10* (AT2G18290), *APC8* (AT3G48150), *CDC20.1* (AT4G33270), *CCS52A2* (AT4G11920), *CCS52B* (AT5G13840), *DMC1* (AT3G22880), *HTR2* (AT1G09200), and *ACTIN7* (AT5G09810).

## Supplementary Material

koaf089_Supplementary_Data

## Data Availability

The data underlying this article are available in the article and in its online Supplementary material.

## Supplementary data

The following materials are available in the online version of this article.

**Supplementary Figure S1.** AUR1 ubiquitination is increased in *cdc20.1-3* (Supplementary Supports Figs. S1 and S2).

**Supplementary Figure S2.** Overexpression of AUR1K102A causes imbalanced meiotic chromosome segregation and aneuploidy (Supplementary Supports Fig. S1).

**Supplementary Figure S3.** APC/C promotes AUR1 degradation (Supplementary Supports Fig. S2).

**Supplementary Figure S4.** AUR1 is polyubiquitinated and APC/C promotes AUR1 degradation via the 26S proteasome (Supplementary Supports Fig. S2).

**Supplementary Figure S5.** AUR1 interacts with CCS52A2/B in D-box-dependent manner (Supplementary Supports Figs. S4 and S5).

**Supplementary Figure S6.** Overexpression of CCS52A2/B weakens the interaction between CDC20.1 and APC8 (Supplementary Supports Figs. S4 and S5).

**Supplementary Figure S7.**  *CCS52A2* is indispensable for vegetative development, fertility, and meiosis (Supplementary Supports Fig. S6).

**Supplementary Figure S8.** CCS52A2 does not affect SYN1 loading and H3S10ph signal is enhanced in the *ccs52* mutant (Supplementary Supports Figs. S6 and S5F).

**Supplementary Figure S9.** Genetic analyses of *APC8*, coactivator *CCS52/CDC20.1*, and *AUR1* during meiotic chromosome segregation (Supplementary Supports Fig. S7; Figs. S1 and S6).

**Supplementary Table S1.** The differentially ubiquitinated proteins identified in the ubiquitin-modified proteome.

**Supplementary Table S2.** List of primers used in this study.

**Supplementary data set S1.** Statistical analyses performed in this study.

## Dive Curated Terms

The following phenotypic, genotypic, and functional terms are of significance to the work described in this paper:

SYN1 Gramene: AT5G05490
SYN1 Araport: AT5G05490

CCS52A2 Gramene: AT4G11920
CCS52A2 Araport: AT4G11920

APC8 Gramene: AT3G48150
APC8 Araport: AT3G48150

DAPI CHEBI: CHEBI:51231

ZYP1 Gramene: AT1G22275; AT1G22260
ZYP1 Araport: AT1G22275; AT1G22260

CENH3 Gramene: AT1G01370
CENH3 Araport: AT1G01370

## References

[koaf089-B1] Aboelenain M, Schindler K. Aurora kinase B inhibits aurora kinase A to control maternal mRNA translation in mouse oocytes. Development. 2021:148(21):dev199560. 10.1242/dev.199560 34636397 PMC8602942

[koaf089-B2] Ballmer D, Lou HJ, Ishii M, Turk BE, Akiyoshi B. Aurora B controls anaphase onset and error-free chromosome segregation in trypanosomes. J Cell Biol. 2024:223(11):e202401169. 10.1083/jcb.202401169 39196069 PMC11354203

[koaf089-B3] Baloban M, Vanstraelen M, Tarayre S, Reuzeau C, Cultrone A, Mergaert P, Kondorosi E. Complementary and dose-dependent action of AtCCS52A isoforms in endoreduplication and plant size control. New Phytol. 2013:198(4):1049–1059. 10.1111/nph.12216 23528034

[koaf089-B4] Berthezene J, Reyes C, Li T, Coulon S, Bernard P, Gachet Y, Tournier S. Aurora B and condensin are dispensable for chromosome arm and telomere separation during meiosis II. Mol Biol Cell. 2020:31(9):889–905. 10.1091/mbc.E20-01-0021 32101485 PMC7185977

[koaf089-B5] Blanco MA, Pelloquin L, Moreno S. Fission yeast mfr1 activates APC and coordinates meiotic nuclear division with sporulation. J Cell Sci. 2001:114(11):2135–2143. 10.1242/jcs.114.11.2135 11493649

[koaf089-B6] Blengini CS, Ibrahimian P, Vaskovicova M, Drutovic D, Solc P, Schindler K. Aurora kinase A is essential for meiosis in mouse oocytes. PLoS Genet. 2021:17(4):e1009327. 10.1371/journal.pgen.1009327 33901174 PMC8102010

[koaf089-B7] Blengini CS, Vaskovicova M, Schier J, Drutovic D, Schindler K. Spatio-temporal requirements of Aurora kinase A in mouse oocyte meiotic spindle building. iScience. 2024:27(8):110451. 10.1016/j.isci.2024.110451 39081293 PMC11284559

[koaf089-B8] Buonomo SBC, Clyne RK, Fuchs J, Loidl J, Uhlmann F, Nasmyth K. Disjunction of homologous chromosomes in meiosis I depends on proteolytic cleavage of the meiotic cohesin Rec8 by separin. Cell. 2000:103(3):387–398. 10.1016/S0092-8674(00)00131-8 11081626

[koaf089-B9] Bustin SA . Why the need for qPCR publication guidelines? The case for MIQE. Methods. 2010:50(4):217–226. 10.1016/j.ymeth.2009.12.006 20025972

[koaf089-B10] Castro A, Arlot-Bonnemains Y, Vigneron S, Labbe JC, Prigent C, Lorca T. APC/fizzy-related targets Aurora-A kinase for proteolysis. EMBO Rep. 2002:3(5):457–462. 10.1093/embo-reports/kvf095 11964384 PMC1084108

[koaf089-B11] Chang L, Zhang Z, Yang J, McLaughlin SH, Barford D. Atomic structure of the APC/C and its mechanism of protein ubiquitination. Nature. 2015:522(7557):450–454. 10.1038/nature14471 26083744 PMC4608048

[koaf089-B12] Chelysheva L, Grandont L, Vrielynck N, le Guin S, Mercier R, Grelon M. An easy protocol for studying chromatin and recombination protein dynamics during *Arabidopsis thaliana* meiosis: immunodetection of cohesins, histones and MLH1. Cytogenet Genome Res. 2010:129(1–3):143–153. 10.1159/000314096 20628250

[koaf089-B13] Chen H, Zou Y, Shang Y, Lin H, Wang Y, Cai R, Tang X, Zhou JM. Firefly luciferase complementation imaging assay for protein-protein interactions in plants. Plant Physiol. 2008:146(2):368–376. 10.1104/pp.107.111740 18065554 PMC2245818

[koaf089-B14] Chikashige Y, Yamane M, Okamasa K, Osakada H, Tsutsumi C, Nagahama Y, Fukuta N, Haraguchi T, Hiraoka Y. Fission yeast APC/C activators Slp1 and Fzr1 sequentially trigger two consecutive nuclear divisions during meiosis. FEBS Lett. 2017:591(7):1029–1040. 10.1002/1873-3468.12612 28245054

[koaf089-B15] Chu T, Henrion G, Haegeli V, Strickland S. *Cortex*, a Drosophila gene required to complete oocyte meiosis, is a member of the Cdc20/fizzy protein family. Genesis. 2001:29(3):141–152. 10.1002/gene.1017 11252055

[koaf089-B16] Chunduri NK, Storchova Z. The diverse consequences of aneuploidy. Nat Cell Biol. 2019:21(1):54–62. 10.1038/s41556-018-0243-8 30602769

[koaf089-B17] Clough SJ, Bent AF. Floral dip: a simplified method for *Agrobacterium*-mediated transformation of *Arabidopsis thaliana*. Plant J. 1998:16(6):735–743. 10.1046/j.1365-313x.1998.00343.x 10069079

[koaf089-B18] Cromer L, Jolivet S, Horlow C, Chelysheva L, Heyman J, De Jaeger G, Koncz C, De Veylder L, Mercier R. Centromeric cohesion is protected twice at meiosis, by SHUGOSHINs at anaphase I and by PATRONUS at interkinesis. Curr Biol. 2013:23(21):2090–2099. 10.1016/j.cub.2013.08.036 24206843

[koaf089-B19] Cromer L, Jolivet S, Singh DK, Berthier F, De Winne N, De Jaeger G, Komaki S, Prusicki MA, Schnittger A, Guerois R, et al Patronus is the elusive plant securin, preventing chromosome separation by antagonizing separase. Proc Natl Acad Sci U S A. 2019:116(32):16018–16027. 10.1073/pnas.1906237116 31324745 PMC6690013

[koaf089-B20] da Fonseca PC, Kong EH, Zhang Z, Schreiber A, Williams MA, Morris EP, Barford D. Structures of APC/C^Cdh1^ with substrates identify Cdh1 and Apc10 as the D-box co-receptor. Nature. 2011:470(7333):274–278. 10.1038/nature09625 21107322 PMC3037847

[koaf089-B21] Demidov D, Lermontova I, Weiss O, Fuchs J, Rutten T, Kumke K, Sharbel TF, Van Damme D, De Storme N, Geelen D, et al Altered expression of Aurora kinases in Arabidopsis results in aneu- and polyploidization. Plant J. 2014:80(3):449–461. 10.1111/tpj.12647 25146886

[koaf089-B22] Demidov D, Van Damme D, Geelen D, Blattner FR, Houben A. Identification and dynamics of two classes of aurora-like kinases in Arabidopsis and other plants. Plant Cell. 2005:17(3):836–848. 10.1105/tpc.104.029710 15722465 PMC1069702

[koaf089-B23] Deng X, Peng FL, Tang X, Lee YJ, Lin HH, Liu B. The Arabidopsis BUB1/MAD3 family protein BMF3 requires BUB3.3 to recruit CDC20 to kinetochores in spindle assembly checkpoint signaling. Proc Natl Acad Sci U S A. 2024b:121(12):e2322677121. 10.1073/pnas.2322677121 38466841 PMC10963012

[koaf089-B24] Deng X, Xiao Y, Tang X, Liu B, Lin H. Arabidopsis α-Aurora kinase plays a role in cytokinesis through regulating MAP65-3 association with microtubules at phragmoplast midzone. Nat Commun. 2024a:15(1):3779. 10.1038/s41467-024-48238-9 38710684 PMC11074315

[koaf089-B25] Ditchfield C, Johnson VL, Tighe A, Ellston R, Haworth C, Johnson T, Mortlock A, Keen N, Taylor SS. Aurora B couples chromosome alignment with anaphase by targeting BubR1, Mad2, and Cenp-E to kinetochores. J Cell Biol. 2003:161(2):267–280. 10.1083/jcb.200208091 12719470 PMC2172902

[koaf089-B26] Floyd S, Pines J, Lindon C. APC/C^Cdh1^ targets aurora kinase to control reorganization of the mitotic spindle at anaphase. Curr Biol. 2008:18(21):1649–1658. 10.1016/j.cub.2008.09.058 18976910

[koaf089-B27] Fülöp K, Tarayre S, Kelemen Z, Horváth G, Kevei Z, Nikovics K, Bakó L, Brown S, Kondorosi A, Kondorosi E. *Arabidopsis* anaphase-promoting complexes: multiple activators and wide range of substrates might keep APC perpetually busy. Cell Cycle. 2005:4(8):1084–1092. 10.4161/cc.4.8.1856 15970679

[koaf089-B28] Garcia-Cano E, Zaltsman A, Citovsky V. Assaying proteasomal degradation in a cell-free system in plants. J Vis Exp. 2014:85:51293. 10.3791/51293 PMC4090386 24747194

[koaf089-B29] Hauf S, Biswas A, Langegger M, Kawashima SA, Tsukahara T, Watanabe Y. Aurora controls sister kinetochore mono-orientation and homolog bi-orientation in meiosis-I. EMBO J. 2007:26(21):4475–4486. 10.1038/sj.emboj.7601880 17932486 PMC2034495

[koaf089-B30] Herbert M, Levasseur M, Homer H, Yallop K, Murdoch A, McDougall A. Homologue disjunction in mouse oocytes requires proteolysis of securin and cyclin B1. Nat Cell Biol. 2003:5(11):1023–1025. 10.1038/ncb1062 14593421

[koaf089-B31] Hjerpe R, Aillet F, Lopitz-Otsoa F, Lang V, England P, Rodriguez MS. Efficient protection and isolation of ubiquitylated proteins using tandem ubiquitin-binding entities. EMBO Rep. 2009:10(11):1250–1258. 10.1038/embor.2009.192 19798103 PMC2775171

[koaf089-B32] Holt JE, Lane SI, Jennings P, Garcia-Higuera I, Moreno S, Jones KT. APC^FZR1^ prevents nondisjunction in mouse oocytes by controlling meiotic spindle assembly timing. Mol Biol Cell. 2012:23(20):3970–3981. 10.1091/mbc.e12-05-0352 22918942 PMC3469513

[koaf089-B33] Holt JE, Pye V, Boon E, Stewart JL, Garcia-Higuera I, Moreno S, Rodriguez R, Jones KT, McLaughlin EA. The APC/C activator FZR1 is essential for meiotic prophase I in mice. Development. 2014:141(6):1354–1365. 10.1242/dev.104828 24553289

[koaf089-B34] Holt JE, Tran SM, Stewart JL, Minahan K, Garcia-Higuera I, Moreno S, Jones KT. The APC/C activator FZR1 coordinates the timing of meiotic resumption during prophase I arrest in mammalian oocytes. Development. 2011:138(5):905–913. 10.1242/dev.059022 21270054

[koaf089-B35] Homer H, Gui L, Carroll J. A spindle assembly checkpoint protein functions in prophase I arrest and prometaphase progression. Science. 2009:326(5955):991–994. 10.1126/science.1175326 19965510 PMC3428834

[koaf089-B36] Huang J, Wang C, Wang H, Lu P, Zheng B, Ma H, Copenhaver GP, Wang Y. Meiocyte-specific and AtSPO11-1-dependent small RNAs and their association with meiotic gene expression and recombination. Plant Cell. 2019:31(2):444–464. 10.1105/tpc.18.00511 30674694 PMC6447014

[koaf089-B37] Jeganathan K, Malureanu L, Baker DJ, Abraham SC, van Deursen JM. Bub1 mediates cell death in response to chromosome missegregation and acts to suppress spontaneous tumorigenesis. J Cell Biol. 2007:179(2):255–267. 10.1083/jcb.200706015 17938250 PMC2064762

[koaf089-B38] Jin F, Hamada M, Malureanu L, Jeganathan KB, Zhou W, Morbeck DE, van Deursen JM. Cdc20 is critical for meiosis I and fertility of female mice. PLoS Genet. 2010:6(9):e1001147. 10.1371/journal.pgen.1001147 20941357 PMC2947999

[koaf089-B39] Jonak K, Zagoriy I, Oz T, Graf P, Rojas J, Mengoli V, Zachariae W. APC/C-Cdc20 mediates deprotection of centromeric cohesin at meiosis II in yeast. Cell Cycle. 2017:16(12):1145–1152. 10.1080/15384101.2017.1320628 28514186 PMC5499901

[koaf089-B40] Jordan P, Copsey A, Newnham L, Kolar E, Lichten M, Hoffmann E. Ipl1/Aurora B kinase coordinates synaptonemal complex disassembly with cell cycle progression and crossover formation in budding yeast meiosis. Genes Dev. 2009:23(18):2237–2251. 10.1101/gad.536109 19759266 PMC2751982

[koaf089-B41] Kawabe A, Matsunaga S, Nakagawa K, Kurihara D, Yoneda A, Hasezawa S, Uchiyama S, Fukui K. Characterization of plant Aurora kinases during mitosis. Plant Mol Biol. 2005:58(1):1–13. 10.1007/s11103-005-3454-x 16028112

[koaf089-B42] Kevei Z, Baloban M, Da Ines O, Tiricz H, Kroll A, Regulski K, Mergaert P, Kondorosi E. Conserved CDC20 cell cycle functions are carried out by two of the five isoforms in *Arabidopsis thaliana*. PLoS One. 2011:6(6):e20618. 10.1371/journal.pone.0020618 21687678 PMC3110789

[koaf089-B43] Kim J, Ishiguro K, Nambu A, Akiyoshi B, Yokobayashi S, Kagami A, Ishiguro T, Pendas AM, Takeda N, Sakakibara Y, et al Meikin is a conserved regulator of meiosis-I-specific kinetochore function. Nature. 2015:517(7535):466–471. 10.1038/nature14097 25533956

[koaf089-B44] Kimata Y, Baxter JE, Fry AM, Yamano H. A role for the Fizzy/Cdc20 family of proteins in activation of the APC/C distinct from substrate recruitment. Mol Cell. 2008:32(4):576–583. 10.1016/j.molcel.2008.09.023 19026787

[koaf089-B45] Komaki S, Takeuchi H, Hamamura Y, Heese M, Hashimoto T, Schnittger A. Functional analysis of the plant chromosomal passenger complex. Plant Physiol. 2020:183(4):1586–1599. 10.1104/pp.20.00344 32461300 PMC7401102

[koaf089-B46] Lin YN, Jiang CK, Cheng ZK, Wang DH, Shen LP, Xu C, Xu ZH, Bai SN. Rice cell division cycle 20s are required for faithful chromosome segregation and cytokinesis during meiosis. Plant Physiol. 2022:188(2):1111–1128. 10.1093/plphys/kiab543 34865119 PMC8825277

[koaf089-B47] Liu L, Zhang Y, Tang S, Zhao Q, Zhang Z, Zhang H, Dong L, Guo H, Xie Q. An efficient system to detect protein ubiquitination by agroinfiltration in *Nicotiana benthamiana*. Plant J. 2010:61(5):893–903. 10.1111/j.1365-313X.2009.04109.x 20015064

[koaf089-B48] Liu Y, Ye W, Li B, Zhou X, Cui Y, Running MP, Liu K. CCS52A2/FZR1, a cell cycle regulator, is an essential factor for shoot apical meristem maintenance in *Arabidopsis thaliana*. BMC Plant Biol. 2012:12(1):135. 10.1186/1471-2229-12-135 22873486 PMC3534500

[koaf089-B49] Ma C, Cummings C, Liu XJ. Biphasic activation of Aurora-A kinase during the meiosis I- meiosis II transition in *Xenopus oocytes*. Mol Cell Biol. 2003:23(5):1703–1716. 10.1128/MCB.23.5.1703-1716.2003 12588989 PMC151708

[koaf089-B50] Meyer HJ, Rape M. Enhanced protein degradation by branched ubiquitin chains. Cell. 2014:157(4):910–921. 10.1016/j.cell.2014.03.037 24813613 PMC4028144

[koaf089-B51] Musacchio A, Salmon ED. The spindle-assembly checkpoint in space and time. Nat Rev Mol Cell Biol. 2007:8(5):379–393. 10.1038/nrm2163 17426725

[koaf089-B52] Nagaoka SI, Hodges CA, Albertini DF, Hunt PA. Oocyte-specific differences in cell-cycle control create an innate susceptibility to meiotic errors. Curr Biol. 2011:21(8):651–657. 10.1016/j.cub.2011.03.003 21497085 PMC3225230

[koaf089-B53] Nguyen HG, Chinnappan D, Urano T, Ravid K. Mechanism of Aurora-B degradation and its dependency on intact KEN and A-boxes: identification of an aneuploidy-promoting property. Mol Cell Biol. 2005:25(12):4977–4992. 10.1128/MCB.25.12.4977-4992.2005 15923616 PMC1140599

[koaf089-B54] Nikalayevich E, El Jailani S, Dupre A, Cladiere D, Gryaznova Y, Fosse C, Buffin E, Touati SA, Wassmann K. Aurora B/C-dependent phosphorylation promotes Rec8 cleavage in mammalian oocytes. Curr Biol. 2022:32(10):2281–2290.e4. 10.1016/j.cub.2022.03.041 35385691

[koaf089-B55] Niu B, Wang L, Zhang L, Ren D, Ren R, Copenhaver GP, Ma H, Wang Y. Arabidopsis *Cell Division Cycle 20.1* is required for normal meiotic spindle assembly and chromosome segregation. Plant Cell. 2015:27(12):3367–3382. 10.1105/tpc.15.00834 26672070 PMC4707457

[koaf089-B56] Ostapenko D, Solomon MJ. APC^Cdh1^-mediated degradation of Cdh1 is necessary for faithful meiotic chromosome segregation in *S. cerevisiae*. bioRxiv 358655. 10.1101/358655, 28 June 2018, preprint: not peer reviewed.

[koaf089-B57] Papini D, Levasseur MD, Higgins JMG. The Aurora B gradient sustains kinetochore stability in anaphase. Cell Rep. 2021:37(6):109818. 10.1016/j.celrep.2021.109818 34758321 PMC8595645

[koaf089-B58] Pesin JA, Orr-Weaver TL. Regulation of APC/C activators in mitosis and meiosis. Annu Rev Cell Dev Biol. 2008:24(1):475–499. 10.1146/annurev.cellbio.041408.115949 18598214 PMC4070676

[koaf089-B59] Peter M, Castro A, Lorca T, Le Peuch C, Magnaghi-Jaulin L, Doree M, Labbe JC. The APC is dispensable for first meiotic anaphase in *Xenopus* oocytes. Nat Cell Biol. 2001:3(1):83–87. 10.1038/35050607 11146630

[koaf089-B60] Peterson R, Slovin JP, Chen C. A simplified method for differential staining of aborted and non-aborted pollen grains. Int J Plant Biol Res. 2010:1(2):e13. 10.4081/pb.2010.e13

[koaf089-B61] Rankin S, Ayad NG, Kirschner MW. Sororin, a substrate of the anaphase-promoting complex, is required for sister chromatid cohesion in vertebrates. Mol Cell. 2005:18(2):185–200. 10.1016/j.molcel.2005.03.017 15837422

[koaf089-B62] Rattani A, Ballesteros Mejia R, Roberts K, Roig MB, Godwin J, Hopkins M, Eguren M, Sanchez-Pulido L, Okaz E, Ogushi S, et al APC/C^Cdh1^ enables removal of Shugoshin-2 from the arms of bivalent chromosomes by moderating Cyclin-dependent kinase activity. Curr Biol. 2017:27(10):1462–1476.e5. 10.1016/j.cub.2017.04.023 28502659 PMC5457479

[koaf089-B63] Reis A, Madgwick S, Chang HY, Nabti I, Levasseur M, Jones KT. Prometaphase APC^cdh1^ activity prevents non-disjunction in mammalian oocytes. Nat Cell Biol. 2007:9(10):1192–1198. 10.1038/ncb1640 17891138 PMC2323444

[koaf089-B64] Ricke RM, Jeganathan KB, van Deursen JM. Bub1 overexpression induces aneuploidy and tumor formation through Aurora B kinase hyperactivation. J Cell Biol. 2011:193(6):1049–1064. 10.1083/jcb.201012035 21646403 PMC3115799

[koaf089-B65] Ruan W, Guo M, Wang X, Guo Z, Xu Z, Xu L, Zhao H, Sun H, Yan C, Yi K. Two RING-finger ubiquitin E3 ligases regulate the degradation of SPX4, an internal phosphate sensor, for phosphate homeostasis and signaling in rice. Mol Plant. 2019:12(8):1060–1074. 10.1016/j.molp.2019.04.003 31002982

[koaf089-B66] Schapira M, Tyers M, Torrent M, Arrowsmith CH. WD40 repeat domain proteins: a novel target class? Nat Rev Drug Discov. 2017:16(11):773–786. 10.1038/nrd.2017.179 29026209 PMC5975957

[koaf089-B67] Schmittgen TD, Livak KJ. Analyzing real-time PCR data by the comparative C(T) method. Nat Protoc. 2008:3(6):1101–1108. 10.1038/nprot.2008.73 18546601

[koaf089-B68] Sen O, Harrison JU, Burroughs NJ, McAinsh AD. Kinetochore life histories reveal an Aurora-B-dependent error correction mechanism in anaphase. Dev Cell. 2021:56(22):3082–3099.e5. 10.1016/j.devcel.2021.10.007 34758290 PMC8629432

[koaf089-B69] Shrestha RL, Conti D, Tamura N, Braun D, Ramalingam RA, Cieslinski K, Ries J, Draviam VM. Aurora-B kinase pathway controls the lateral to end-on conversion of kinetochore-microtubule attachments in human cells. Nat Commun. 2017:8(1):150. 10.1038/s41467-017-00209-z 28751710 PMC5532248

[koaf089-B70] Siomos MF, Badrinath A, Pasierbek P, Livingstone D, White J, Glotzer M, Nasmyth K. Separase is required for chromosome segregation during meiosis I in *Caenorhabditis elegans*. Curr Biol. 2001:11(23):1825–1835. 10.1016/S0960-9822(01)00588-7 11728305

[koaf089-B71] Sivakumar S, Gorbsky GJ. Spatiotemporal regulation of the anaphase-promoting complex in mitosis. Nat Rev Mol Cell Biol. 2015:16(2):82–94. 10.1038/nrm3934 25604195 PMC4386896

[koaf089-B72] Sun L, Bai S, Chen JL, Deng DJ, Luo ZQ, Wang Y, Jin QW. A dual mechanism of APC/C inhibition by MAP kinases. bioRxiv 485959. 10.1101/2022.04.01.485959, 3 April 2022, preprint: not peer reviewed.

[koaf089-B73] Swain JE, Ding J, Wu J, Smith GD. Regulation of spindle and chromatin dynamics during early and late stages of oocyte maturation by aurora kinases. Mol Hum Reprod. 2008:14(5):291–299. 10.1093/molehr/gan015 18353803 PMC2408935

[koaf089-B74] Taguchi S, Honda K, Sugiura K, Yamaguchi A, Furukawa K, Urano T. Degradation of human Aurora-A protein kinase is mediated by hCdh1. FEBS Lett. 2002:519(1–3):59–65. 10.1016/S0014-5793(02)02711-4 12023018

[koaf089-B75] Tanno N, Kuninaka S, Fujimura S, Takemoto K, Okamura K, Takeda N, Araki K, Araki M, Saya H, Ishiguro KI. Phosphorylation of the Anaphase Promoting Complex activator FZR1/CDH1 is required for Meiosis II entry in mouse male germ cell. Sci Rep. 2020:10(1):10094. 10.1038/s41598-020-67116-0 32572094 PMC7308413

[koaf089-B76] Tarayre S, Vinardell JM, Cebolla A, Kondorosi A, Kondorosi E. Two classes of the CDh1-type activators of the anaphase-promoting complex in plants: novel functional domains and distinct regulation. Plant Cell. 2004:16(2):422–434. 10.1105/tpc.018952 14742878 PMC341914

[koaf089-B77] Van Damme D, De Rybel B, Gudesblat G, Demidov D, Grunewald W, De Smet I, Houben A, Beeckman T, Russinova E. Arabidopsis alpha Aurora kinases function in formative cell division plane orientation. Plant Cell. 2011:23(11):4013–4024. 10.1105/tpc.111.089565 22045917 PMC3246319

[koaf089-B78] Wang C, Huang J, Li Y, Zhang J, He C, Li T, Jiang D, Dong A, Ma H, Copenhaver GP, et al DNA polymerase epsilon binds histone H3.1-H4 and recruits MORC1 to mediate meiotic heterochromatin condensation. Proc Natl Acad Sci U S A. 2022:119(43):e2213540119. 10.1073/pnas.2213540119 36260743 PMC9618065

[koaf089-B79] Wang T, Wang H, Lian Q, Jia Q, You C, Copenhaver GP, Wang C, Wang Y. HEI10 is subject to phase separation and mediates RPA1a degradation during meiotic interference-sensitive crossover formation. Proc Natl Acad Sci U S A. 2023:120(52):e2310542120. 10.1073/pnas.2310542120 38134200 PMC10756261

[koaf089-B80] Wang Y, Cheng Z, Lu P, Timofejeva L, Ma H. Molecular cell biology of male meiotic chromosomes and isolation of male meiocytes in *Arabidopsis thaliana*. Methods Mol Biol. 2014:1110:217–230. 10.1007/978-1-4614-9408-9_10 24395259

[koaf089-B81] Wang Y, Sun S, Zhu W, Jia K, Yang H, Wang X. Strigolactone/MAX2-induced degradation of brassinosteroid transcriptional effector BES1 regulates shoot branching. Dev Cell. 2013:27(6):681–688. 10.1016/j.devcel.2013.11.010 24369836

[koaf089-B82] Wellard SR, Schindler K, Jordan PW. Aurora B and C kinases regulate chromosome desynapsis and segregation during mouse and human spermatogenesis. J Cell Sci. 2020:133(23):jcs248831. 10.1242/jcs.248831 33172986 PMC7725601

[koaf089-B83] Willems A, De Veylder L. The plant anaphase-promoting Complex/cyclosome. Annu Rev Cell Dev Biol. 2022:38(1):25–48. 10.1146/annurev-cellbio-120420-092421 35395166

[koaf089-B84] Xu RY, Xu J, Wang L, Niu B, Copenhaver GP, Ma H, Zheng B, Wang Y. The Arabidopsis anaphase-promoting complex/cyclosome subunit 8 is required for male meiosis. New Phytol. 2019:224(1):229–241. 10.1111/nph.16014 31230348 PMC6771777

[koaf089-B85] Xu W, Yu Y, Jing J, Wu Z, Zhang X, You C, Ma H, Copenhaver GP, He Y, Wang Y. SCF^RMF^ mediates degradation of the meiosis-specific recombinase DMC1. Nat Commun. 2023:14(1):5044. 10.1038/s41467-023-40799-5 37598222 PMC10439943

[koaf089-B86] Yang C, Hamamura Y, Sofroni K, Bower F, Stolze SC, Nakagami H, Schnittger A. SWITCH 1/DYAD is a WINGS APART-LIKE antagonist that maintains sister chromatid cohesion in meiosis. Nat Commun. 2019:10(1):1755. 10.1038/s41467-019-09759-w 30988453 PMC6465247

[koaf089-B87] Yao XB, Abrieu A, Zheng Y, Sullivan KF, Cleveland DW. CENP-E forms a link between attachment of spindle microtubules to kinetochores and the mitotic checkpoint. Nat Cell Biol. 2000:2(8):484–491. 10.1038/35019518 10934468

[koaf089-B88] Yoshida S, Kaido M, Kitajima TS. Inherent instability of correct kinetochore-microtubule attachments during meiosis I in oocytes. Dev Cell. 2015:33(5):589–602. 10.1016/j.devcel.2015.04.020 26028219

[koaf089-B89] Zamariola L, Tiang CL, De Storme N, Pawlowski W, Geelen D. Chromosome segregation in plant meiosis. Front Plant Sci. 2014:5:279. 10.3389/fpls.2014.00279 24987397 PMC4060054

[koaf089-B90] Zhao J, Li H, Chen G, Du L, Xu P, Zhang X, Xie M, Cao T, Li H. Aneuploid abortion correlates positively with MAD1 overexpression and miR-125b down-regulation. Mol Cytogenet. 2021:14(1):22. 10.1186/s13039-021-00538-1 33902659 PMC8074413

[koaf089-B91] Zheng B, Chen X, McCormick S. The anaphase-promoting complex is a dual integrator that regulates both microRNA-mediated transcriptional regulation of *cyclin B1* and degradation of cyclin B1 during *Arabidopsis* male gametophyte development. Plant Cell. 2011:23(3):1033–1046. 10.1105/tpc.111.083980 21441434 PMC3082252

[koaf089-B92] Zhong S, Xu Y, Yu C, Zhang X, Li L, Ge H, Ren G, Wang Y, Ma J, Zheng Y, et al Anaphase-promoting complex/cyclosome regulates RdDM activity by degrading DMS3 in *Arabidopsis*. Proc Natl Acad Sci U S A. 2019:116(9):3899–3908. 10.1073/pnas.1816652116 30760603 PMC6397581

