# Supplementary material for: ANAPHASE-PROMOTING COMPLEX/CYCLOSOME coactivators maintain AURORA 1 kinase homeostasis during meiotic chromosome segregation
Source: Plant Cell. 2025 Apr 17;37(6):koaf089. doi: 10.1093/plcell/koaf089 (PMC12164589; doi:10.1093/plcell/koaf089)
Supplement: koaf089_Supplementary_Data [file koaf089_supplementary_data.zip › TPC-2025-0090R1_Supplementary figures.pdf]

Supplementary Figure S1

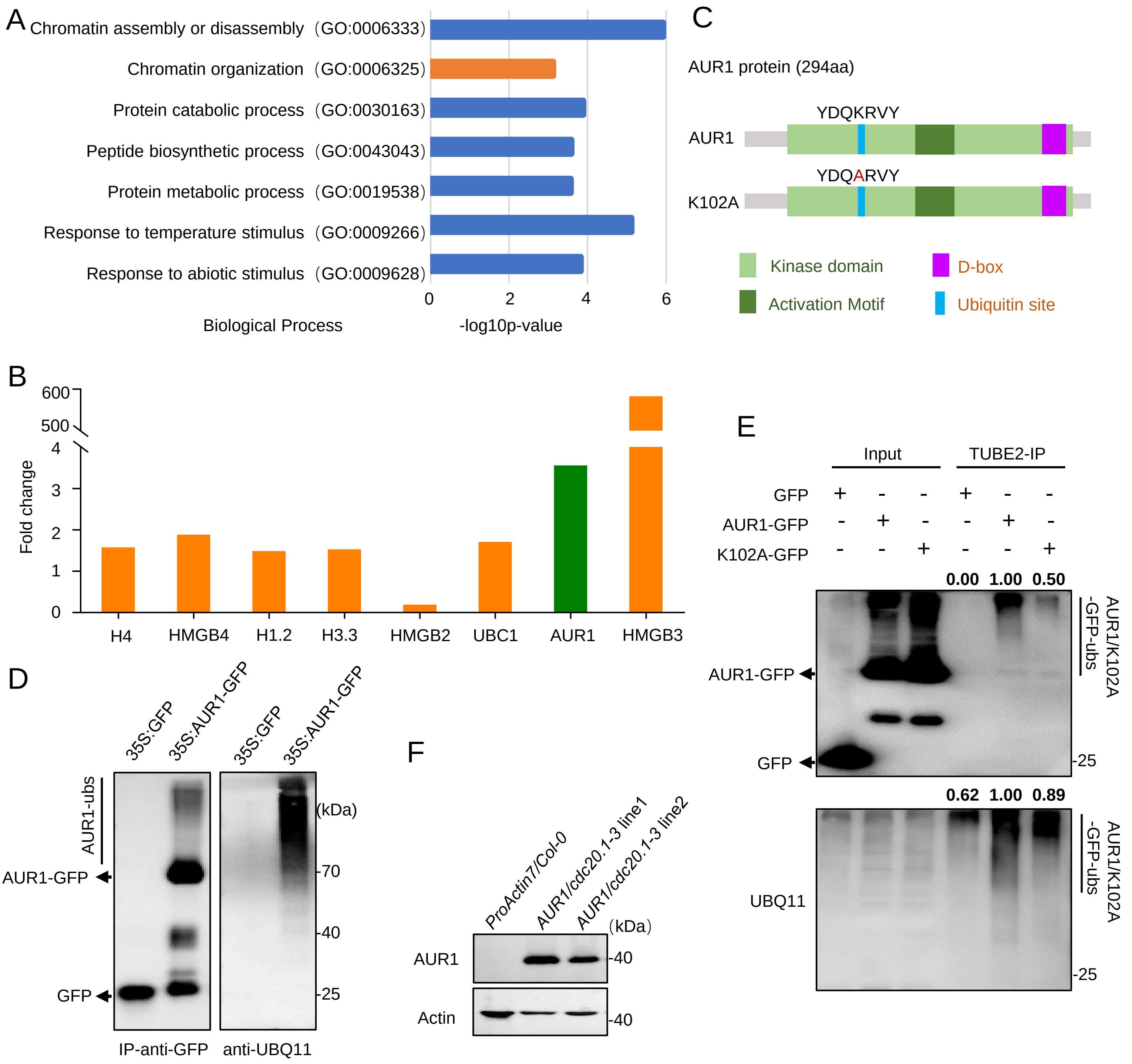

Supplementary Figure S1. AUR1 ubiquitination is increased in *cdc20.1-3*. (Supports Figure 1 and Figure 2).

(A) Enrichment analysis of Gene Ontology (GO) annotations in identified up-regulated 207 ubiquitinated proteins in young inflorescences of *cdc20.1-3* compared to that of Col-0 by IP-MS/MS ubiquitin-modified proteome data. The -log10 (*p* values) of each term are taken as the abscissa. One-tailed statistical overrepresentation test was performed without adjustment for multiple comparisons for GO term analysis. The orange column indicates chromatin organization process.

(B) Histogram of the fold change of chromosome organization related 8 proteins that enrichment in chromosome organization process (GO:0006325) and protein metabolism (GO:0019538).

(C) Schematic diagram of AUR1 protein domains and the identified ubiquitinated site were shown with grass green, green and purple boxes. Ubiquitinated residue in AUR1 identified by LC-MS/MS analysis is shown in blue with amino acid position labeled (K102A).

(D) AUR1 protein is ubiquitinated in *Nicotiana benthamiana* leaves. Total protein was extracted and immunoprecipitated using magnetic beads crosslinking anti-GFP antibody, then immunoblotting using antibodies against GFP (left) and UBQ11 (right) separately to detect AUR1-ubs conjugates. GFP sample was considered as negative controls. The asterisk labels AUR1-GFP target protein. The experiments with three replicates show similar tendency, 6 *Nicotiana benthamiana* strains in each replicate at the same treated time. The molecular weight (kDa) was labeled on the right of the images (same for other blot results).

(E) Ubiquitination of AUR1 is decreased in *AUR1K102A* transgenic plants. Equal amount of transiently expressed AUR1-GFP, K102A-GFP treated with 50 μM MG132 in *Nicotiana benthamiana* leaves were collected. Protein extracts were immunoprecipitated with TUBE2 affinity gel matrix. The immunoprecipitation (IP) samples were analyzed by immunoblotting using anti-GFP antibody (top) and anti-UBQ11 antibody (bottom) to detect the ubiquitinated level of intact AUR1 and AUR1 K102A. The experiments with three replicates show similar tendency, 6 *Nicotiana benthamiana* strains in each replicate at the same treated time, the relative bands intensities of immunoprecipitated proteins normalized to AUR1-GFP were labeled. **(F)** Western blot shows the expression of AUR1 in central inflorescences of three individual plants from two independent lines of *ProActin7:AUR1-FLAG/cdc20.1* transgenic plants (used in Figure 1) and simultaneous expressing FLAG tag driven by the *Actin7* promotor was used as a negative control. Immunoblot using anti-FLAG (top), Actin (bottom) antibodies examined the corresponding proteins.

Supplementary Figure S2

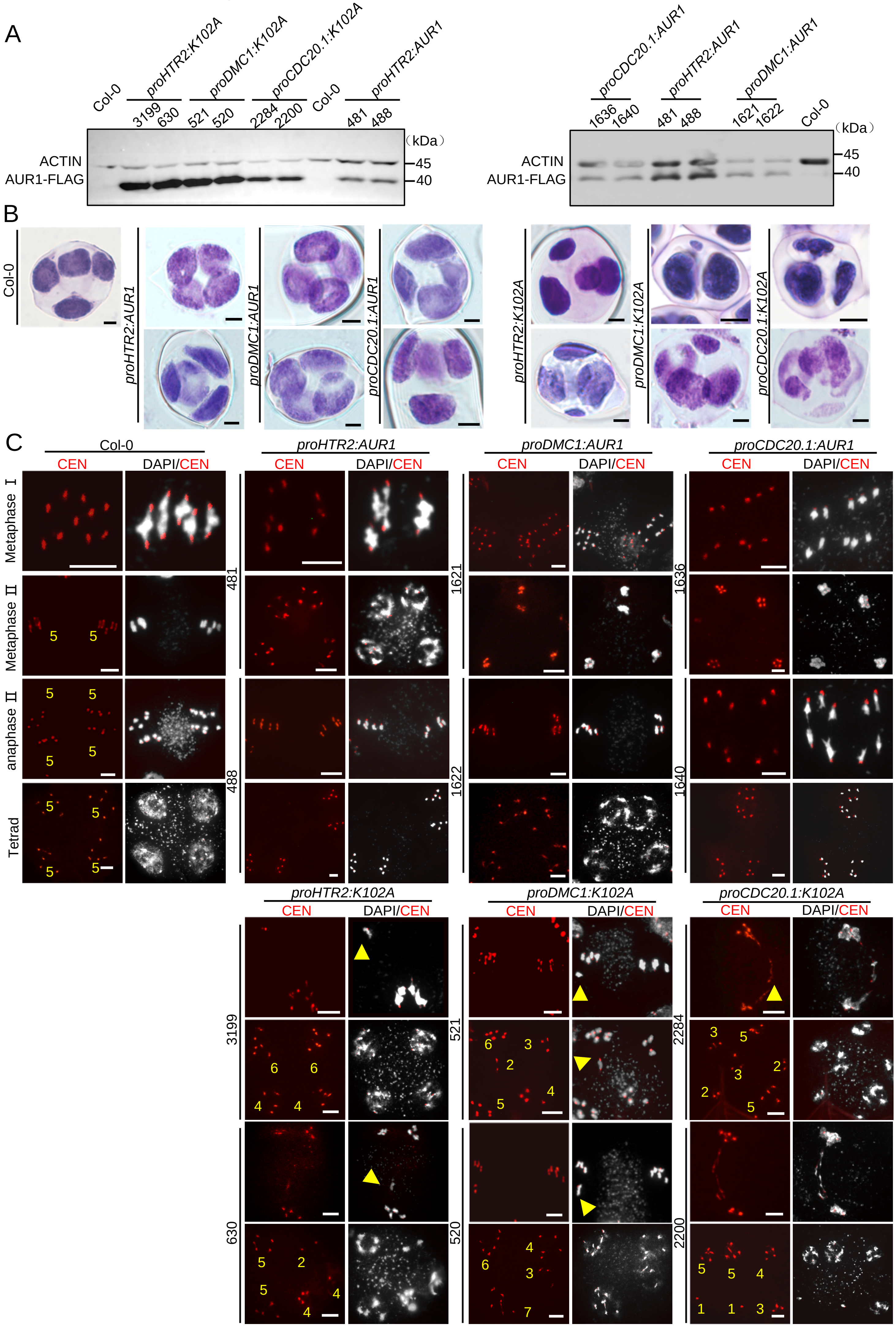

Supplementary Figure S2. Overexpression of AUR1K102A causes imbalanced meiotic chromosome segregation and aneuploidy. (Supports Figure 1).

(A) Intact AUR1 and AUR1K102A-FLAG proteins were expressed in *ProHTR2:AUR1-FLAG/*Col-0 (independent transgenic lines numbered as 481, 488)*, ProDMC1:AUR1-FLAG/*Col-0 (numbered as 1621, 1622), *ProCDC20.1:AUR1-FLAG/*Col-0 (numbered as 1636, 1640), *ProHTR2:AUR1K102A-FLAG/*Col-0 (numbered as 3199, 630), *ProDMC1:AUR1K102A-FLAG/*Col-0 (numbered as 521, 520), *ProCDC20.1:AUR1K102A-FLAG/*Col-0 (numbered as 2284, 2200) transgenic plants (constructs using CDS sequences of *AUR1* or *AUR1K102A* driven under the control of *HTR2*, *DMC1* and *CDC20.1* promoter). Anti-FLAG antibody was used to detect the AUR1 and AUR1K102A protein in anthers from central inflorescences of three individual plants form Col-0, two independent lines of overexpressing *AUR1 or AUR1K102A* transgenic plants, respectively, ACTIN antibody was included as a sample loading control.

(B) Tetrad stage microspores stained with toluidine blue from three individual plants of Col-0 (n = 20) and the above transgenic plants (n = 150 of AUR1 overexpressing plants, n = 150 of AUR1K102A overexpressing plants) in (A). AUR1K102A over-expressing transgenic plants representative polyads and untypical tetrads are shown (14.6%, n = 150 of toluidine blue stained were untypical tetrads). Scale bar, 5 μm.

(C) Meiotic chromosome morphology of Col-0, *AUR1* over-expressing transgenic plants and *AUR1K102A* over-expressing transgenic plants in (A). Meiocytes in more than two independent lines show meiotic chromosome segregation defects in *AUR1K102A* over-expressing transgenic plants. For each meiotic stage in Col-0 and AUR1-overexpressing plants, cells isolated from more than three independent plants were observed with similar meiotic chromosome phenotypes; cells isolated from more than three independent plants of AUR1K102A-overexpressing lines were observed abnormal phenotypes shown in the figures, the number of meiocytes observed were no less than 20. Numbers in the figures indicate the segregated chromosome numbers in male meiosis. Arrowhead marks the bivalent with an abnormal centromere signal. All pictures were taken with a ×100 objective using a fluorescence microscope (zeiss Axio Imager). Scale bar, 5 μm.

Supplementary Figure S3

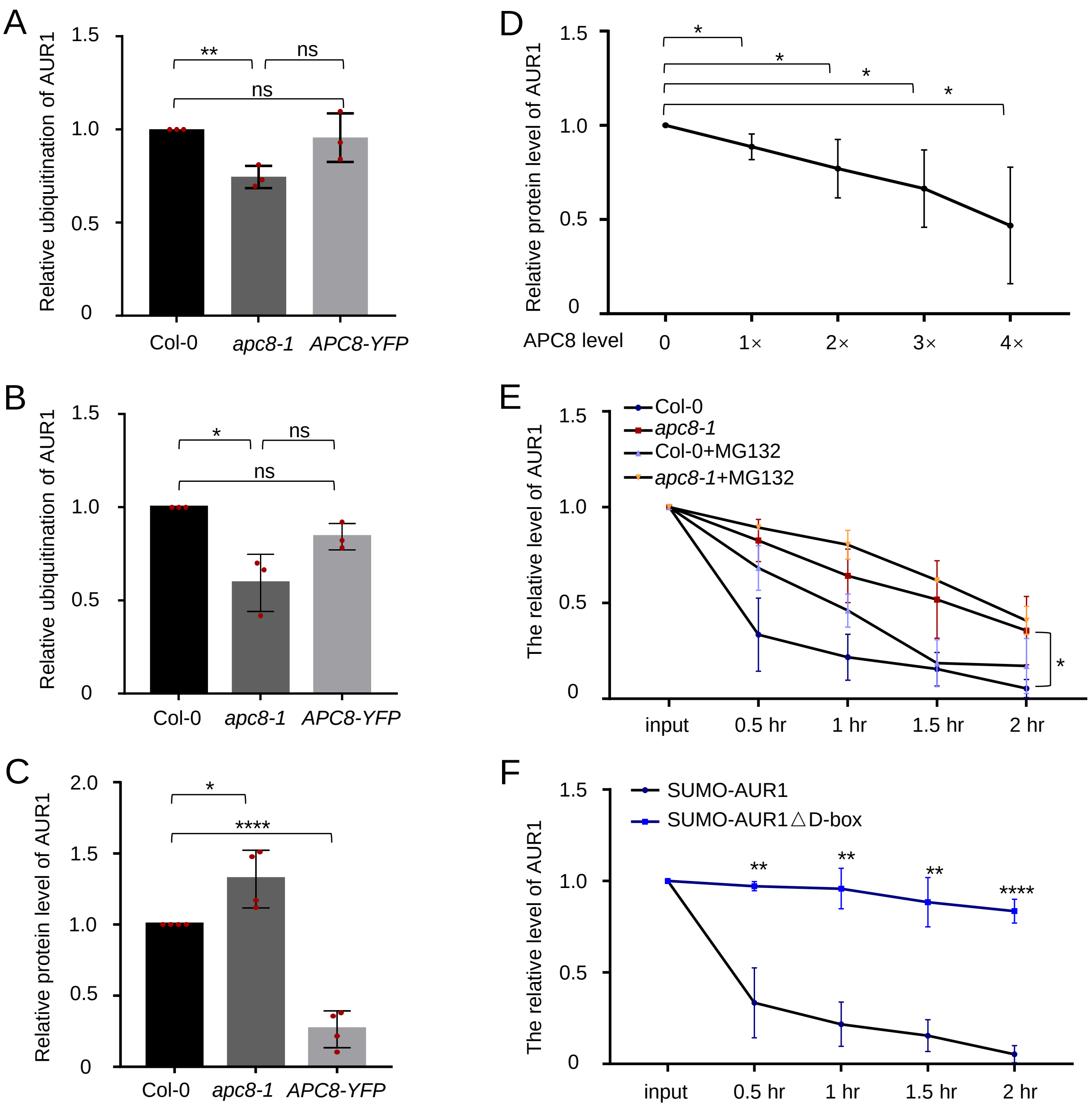

Supplementary Figure S3. APC/C promotes AUR1 degradation. (Supports Figure 2)

(A) to (B) The graphs show the relative ubiquitination levels of AUR1 using FLAG beads-immunoprecipitation (IP) and TUBE2 beads-IP *in vivo* assays in Figure 2A and Figure 2B, respectively. The ubiquitination levels of AUR1 were quantified with Image J software. The value of the samples with Col-0 background was set as 1.0. Data shown are means ± SD of 3 independent replicates (100 inflorescences from 30 plants in each replicate at the same treated time). *apc8-1* vs. Col-0, p = 0.0101; *APC8-YFP* vs. Col-0, p = 0.054; *APC8-YFP* vs. *apc8-1*, p = 0.0654.

(C) The graphs show the relative protein levels of AUR1 in Figure 2C. The value of the samples with Col-0 background was set as 1.0. Data shown are means ± SD of 4 independent replicates (50 inflorescences from 15 plants in each replicate at the same treated time). *apc8-1* vs. Col-0, p = 0.0196; *APC8-YFP* vs. Col-0, ****p < 0.0001.

(D) The broken line graph shows the relative protein levels of AUR1 with the increased APC8 protein in Figure 2D. The value of samples without expressing APC8-MYC was set as 1.0. Data shown are means ± SD of 3 independent replicates (6 *Nicotiana benthamiana* strains in each replicate at the same treated time). APC8-MYC level 1× vs. 0, p = 0.0452; 2× vs. 0, p = 0.049; 3× vs. 0, p = 0.0468; 4× vs. 0, p = 0.041.

(E) The relative remaining protein level of SUMO-AUR1 incubating with equal amount lysate of Col-0 and *apc8-1* with MG132 or without MG132 in Figure 2G. The input value of SUMO-AUR1 was set as 1.0. Data shown are means ± SD of 3 independent replicates without MG132 treatment and 2 independent replicates with MG132 treatment (6 *Nicotiana benthamiana* strains in each replicate at the same treated time). SUMO-AUR1/*apc8-1* vs. SUMO-AUR1/Col-0, p = 0.0205.

(F) The relative remaining protein level of SUMO-AUR1 and SUMO-AUR1△D-box incubating with equal amount lysate of Col-0 for the indicated time in Figure 2H. The input values were set as 1.0, respectively. Data shown are means ± SD of 3 independent replicates (100 inflorescences from 30 Col-0 and 60 *apc8* plants in each replicate at the same treated time).

In (A) to (F), asterisks represent significant differences compared with the input or the Col-0. *p < 0.05, **p < 0.01, ***p < 0.001, ****p < 0.0001, ns, no significance, statistical analysis was performed using the two-tailed Student's *t* test. Source data are provided as a supplementary data set S1.

Supplementary Figure S4

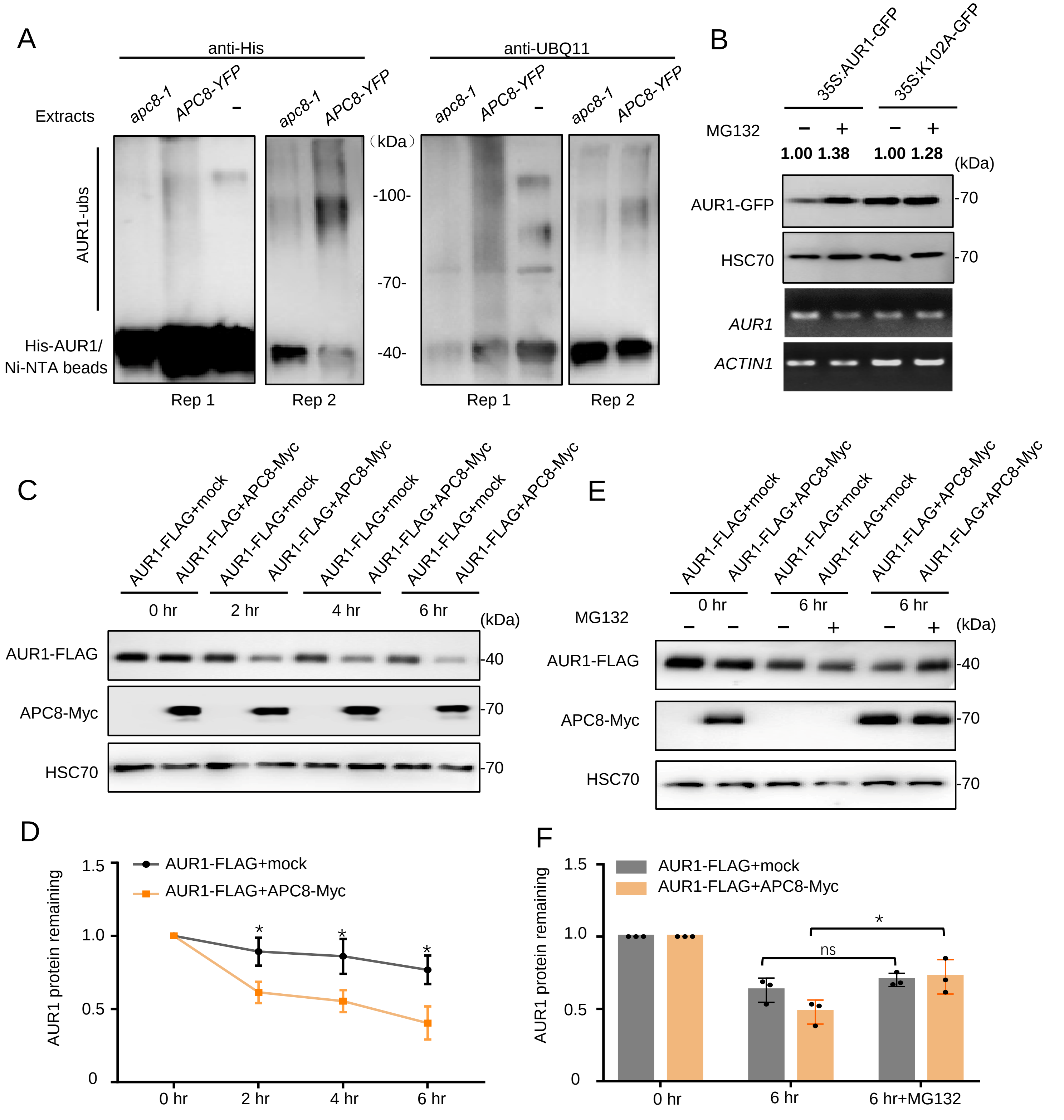

Supplementary Figure S4. AUR1 is polyubiquitinated and APC/C promotes AUR1 degradation via the 26S proteasome. (Supports Figure 2).

(A) APC/C mediates AUR1 ubiquitination using *in vitro* ubiquitination assay. Equal amount of His-tagged recombinant AUR1 proteins bound by Ni-NTA affinity beads were incubated with equal amounts of protein lysates of Col-0, *apc8-1* and *APC8-YFP* plants for 6 hr at 25 ℃. Immunoblot detects ubiquitinated His-AUR1 using anti-His antibody (left two) and anti-UBQ11 antibody (right two). Two biological replicates (Rep) are shown, 100 inflorescences from 25 *apc8-1* and 25 *APC8-YFP* plants in each replicate are used.

(B) The AUR1K102A protein is more stable than intact AUR1 in response to MG132. *Nicotiana benthamiana* leaves expressing AUR1 and K102A proteins were infiltrated with 50 μM MG132 12 hr before harvest and target proteins were detected with GFP antibody. HSC70 antibody (top two) of the house-keeping gene is shown as a loading control. *AUR1* and *ACTIN1* mRNA expression level were detected shown in bottom two lenes. The ratio of the relative density between with (+) or without (−) MG132 treatment (+ MG132/− MG132) are shown. The value of samples without MG132 treatment was set as 1.0. Experiments were conducted in three replicates with similar results (2 *Nicotiana benthamiana* strains in each replicate at the same treated time).

(C) Time-course assay shows that APC8 promotes AUR1 degradation. Total proteins were isolated from *Nicotiana benthamiana* leaves after expressing AUR1-FLAG and mock control or AUR1-FLAG and APC8-Myc together, then extraction incubated at 4 ℃ with gentle shaking. Samples were collected at different time points and AUR1-FLAG was detected by anti-FLAG antibody (top) and APC8-Myc by anti-Myc antibody (middle). Anti-HSC70 (bottom panel) of the house-keeping gene is shown as a loading control.

(D) The statistical analysis of the decay rate of AUR1-FLAG in (C). The level of AUR1 in “AUR-FLAG+mock” and “AUR-FLAG+APC8-Myc” at 0 hr was set as 1.0, respectively. Samples from each group collecting at different time points (2 hr, 4 hr, 6 hr) were plotted versus the level at 0 hr. Three independent biological replicates were performed.

(E) MG132 inhibits or delays AUR1 degradation. The experiment was performed as in (C) with (+) or without (−) addition of MG132 to a final concentration of 50 μM. anti-FLAG (top), Myc (middle) and HSC70 (bottom) antibodies were used to detect corresponding proteins.

(F) The statistical analysis of the decay rate of AUR1-FLAG in (E). The level of AUR1 in “AUR-FLAG+mock” and “AUR-FLAG+APC8-Myc” at 0 hr was set as 1.0, respectively. The level of AUR1 at each group was plotted based on the level at 0 hr. Three biological replicates were performed and showed similar results. The data in (D) and (F) shown as means ± SD (6 *Nicotiana benthamiana* strains in each replicate at the same treated time, respectively). The asterisks indicate significant differences by two-tailed Student’s *t* test (*$p < 0.05$; ns. no significance). Source data are provided as a supplementary data set S1.

Supplementary Figure S5

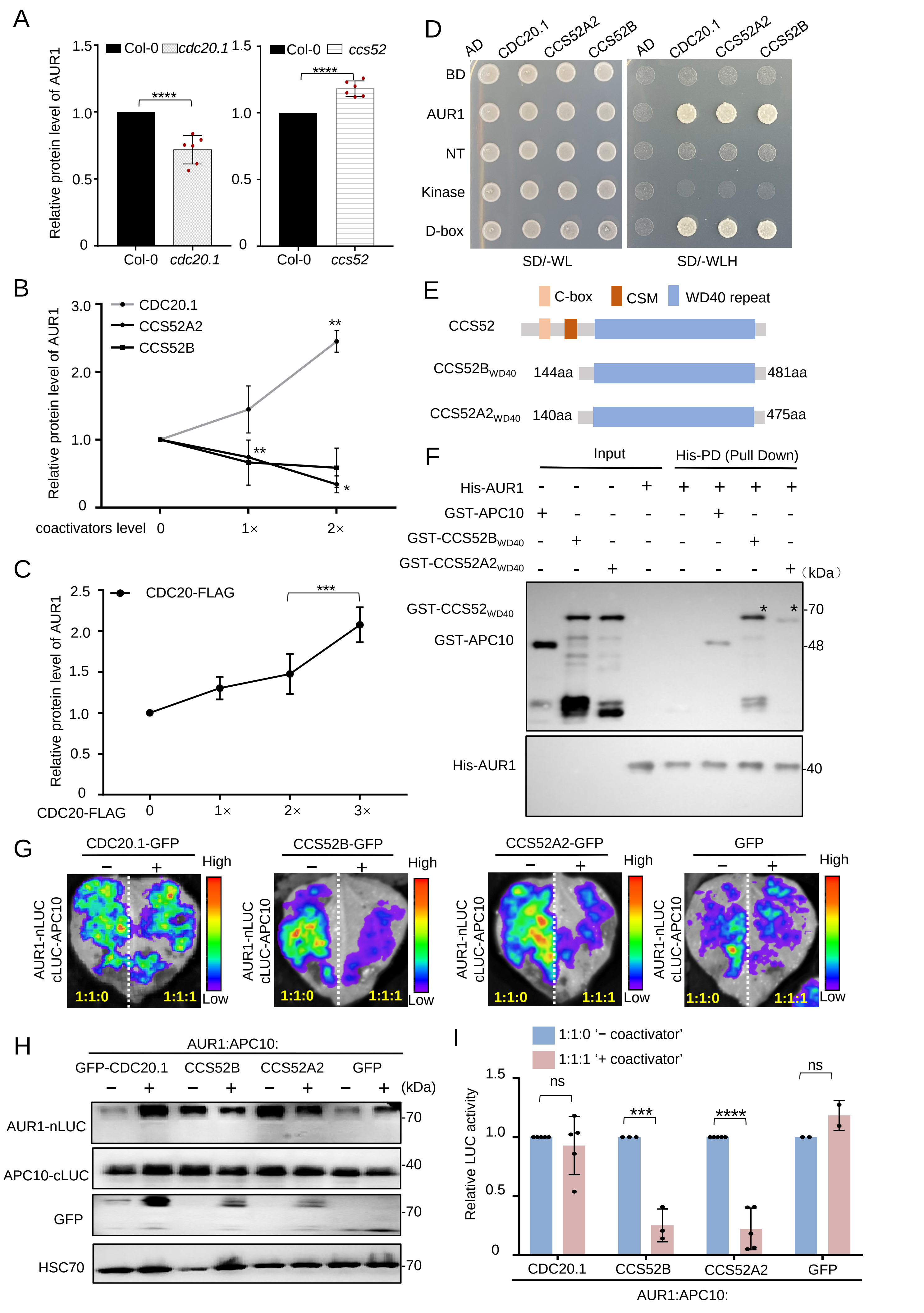

Supplementary Figure S5. AUR1 interacts with CCS52A2/B in D-box dependent manner. (Supports Figure 4 and Figure 5).

(A) The graphs shows the relative protein levels of AUR1 in *cdc20.1-3* and *ccs52a2-1/+ ccs52b-1* relative to Col-0 in Figure 4C and Figure 5F. The value of the samples with Col-0 background was set as 1.0. Data shown are means ± SD of 6 independent replicates (50 inflorescences from 20 plants in each replicate at the same treated time). two-tailed Student's *t* test, *cdc20.1* vs. Col-0, ****p < 0.0001, *ccs52a2-1/+ 52b-1* vs. Col-0, ****p < 0.0001.

(B) The broken line graph shows the relative remaining protein levels of AUR1 with the increased coactivators protein in Figure 4D, 5D and 5E, respectively. The value of samples without expressing coactivators in each independent assay was set as 1.0. Data shown are means ± SD of 2 independent replicates (6 *Nicotiana benthamiana* strains in each replicate at the same treated time). two-tailed Student's *t* test, CDC20.1 2× vs. CDC20.1 0×, p = 0.06, CCS52A2 1× vs. CCS52A2 0×, p = 0.001, CCS52A2 2× vs. CCS52A2 1×, p = 0.0456.

(C) The broken line graph shows the relative remaining protein levels of SUMO-AUR1 with the increased GST-CDC20 and CDC20.1-FLAG in Figure 4E and 4F. The input value of SUMO-AUR1 was set as 1.0. Data shown are means ± SD of 3 independent replicates (6 *Nicotiana benthamiana* strains in each replicate at the same treated time). two-tailed Student's *t* test, CDC20-FLAG 3× vs. 2×, p = 0.0005.

(D) AUR1 interacts with APC/C coactivators through D-box in Y2H. AD and BD empty vectors were used as negative controls. Transformants were plated on synthetic dropout (SD) medium without leucine or tryptophan (SD/−WL), synthetic dropout (SD) medium without leucine, tryptophan, histidine (SD/−WLH) to detect interactions. Experiments were conducted in three replicates with similar results.

(E) to (F) Schematic diagram of full-length CCS52 and deletion derivatives are used in pull down assay. Numbers refer to the positions of the first or last amino acid in the sequences. *In vitro* pull-down assay examines the interaction between AUR1 and $CCS52_{WD40}$. The mixtures of the purified proteins GST-CCS52 were pulled down by His-AUR1 immobilized on the NTA affinity agarose beads were immunoblotted with anti-GST (top two) and anti-His antibodies (bottom). The asterisk (*) indicate the target bands.

(G) Competitive Split-Luciferase Complementation assay examines the effect of CDC20.1 and CCS52A2/B on the interaction between AUR1 and APC10. AUR1 and APC10 were fused N-terminal fragment of Luciferase (nLUC) and C-terminal fragment of Luciferase (cLUC) respectively. The numbers in the bottom corners indicate the volume ratio of AUR1-nLUC, cLUC-APC10 as well as CDC20.1-GFP or CCS52A2/B-GFP, *Nicotiana benthamiana* leaves were then infiltrated with *Agrobacterium tumefaciens* strains harboring with or without CDC20.1 and CCS52A2/B construct designated as Group 1:1:0 '− coactivator' and 1:1:1 '+ coactivator'. Results were captured via fluorescence imager.

(H) Immunoblot detects the expression of relevant proteins in (G). The protein levels of AUR1-nLUC and cLUC-APC10 were detected with anti-LUC antibody (top two), while the protein levels of CCS52-GFP and CDC20.1-GFP were detected with anti-GFP antibody (third panel), anti-HSC70 antibody detect the loading control (bottom).

(I) Statistical analysis of the fluorescence signal from various combinations in (G) using indiGO software. Group 1:1:0 was set as 1.0 then the fluorescence signal intensity in group 1:1:1 were plotted versus the level in Group 1:1:0. In G-I, at least two independent biological replicates were performed with similar tendency (2 *Nicotiana benthamiana* strains in each replicate at the same treated time). The data is shown as means ± SD. The asterisks indicate significant differences compared with the input under the same treatment or compared with Col-0 in A-C, I by two-tailed Student's *t* test. *p < 0.05, **p < 0.01, ***p < 0.001, ****p < 0.0001; ns. no significance. Source data are provided as a supplementary data set S1.

Supplementary Figure S6

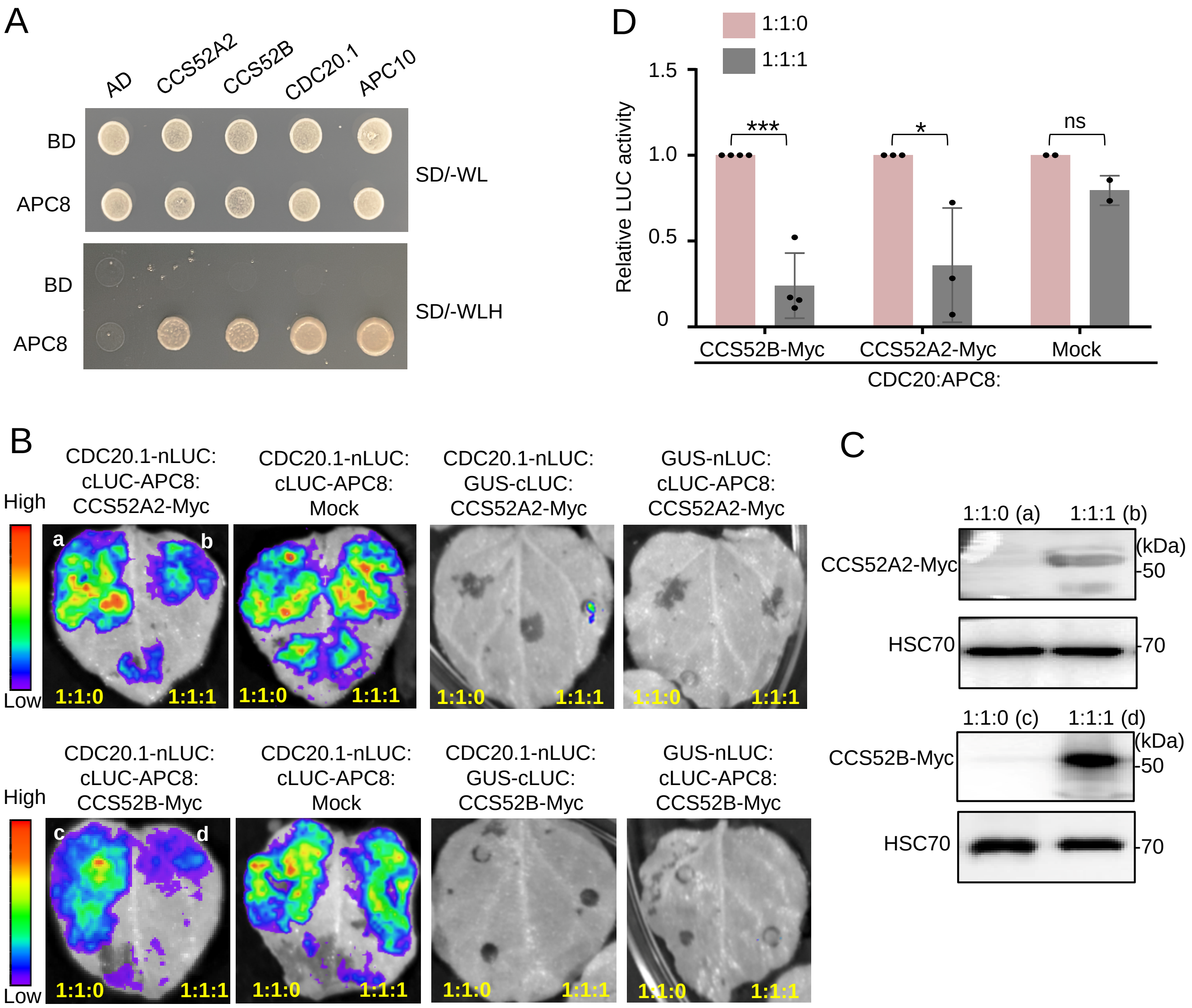

Supplementary Figure S6. Overexpression of CCS52A2/B weakens the interaction between CDC20.1 and APC8. (Supports Figure 4 and Figure 5).

(A) Coactivators CCS52A2/B and CDC20.1 interact with APC8 in Y2H. Transformants were plated on synthetic dropout (SD) medium without leucine or tryptophan (SD/-WL), synthetic dropout (SD) medium without leucine, tryptophan, histidine (SD/-LWH) to detect interactions. Experiments were conducted in three replicates with similar results.

(B) Competitive Split-Luciferase Complementation assay detects the effect of CCS52A2/B on CDC20.1 and APC8 interaction. CCS52A2 (upper four) and CCS52B (bottom four) both decrease the interaction of CDC20.1 with APC8. Yellow numbers in the bottom corner indicate the volume ratio of CDC20.1, APC8 and CCS52A2/B strains infiltrated into the *Nicotiana benthamiana* leaves. The white letter “a-d” in the upper corners indicate infiltrated *Nicotiana benthamiana* region for further examination. The color legend indicates fluorescence signal between CDC20.1 and APC8.

(C) Immunoblot detects the expression of corresponding proteins in (B). The CCS52A2/B protein levels were assessed with anti-Myc antibody, HSC70 was used as the loading control.

(D) Relative luciferase (LUC) activity in (B) were analyzed using indiGO software (Berthhold Technologies). Group 1:1:0 and 1:1:1 correspond to the volume ratio of CDC20.1-nLUC, cLUC-APC8 as well as CCS52A2-Myc or CCS52B-Myc, mock (empty vector with Myc tag) was used as negative control. Group 1:1:0 was set as 1.0 then the fluorescence signal intensity in group 1:1:1 were plotted versus the level in Group 1:1:0. At least two independent biological replicates were performed with similar tendency (3 *Nicotiana benthamiana* strains in each replicate at the same treated time). Data are presented as means ± SD (*p < 0.05, ***p < 0.001; ns. no significance), statistical analysis was performed by two-tailed Student’s *t* test. Source data are provided as a supplementary data set S1.

Supplementary Figure S7

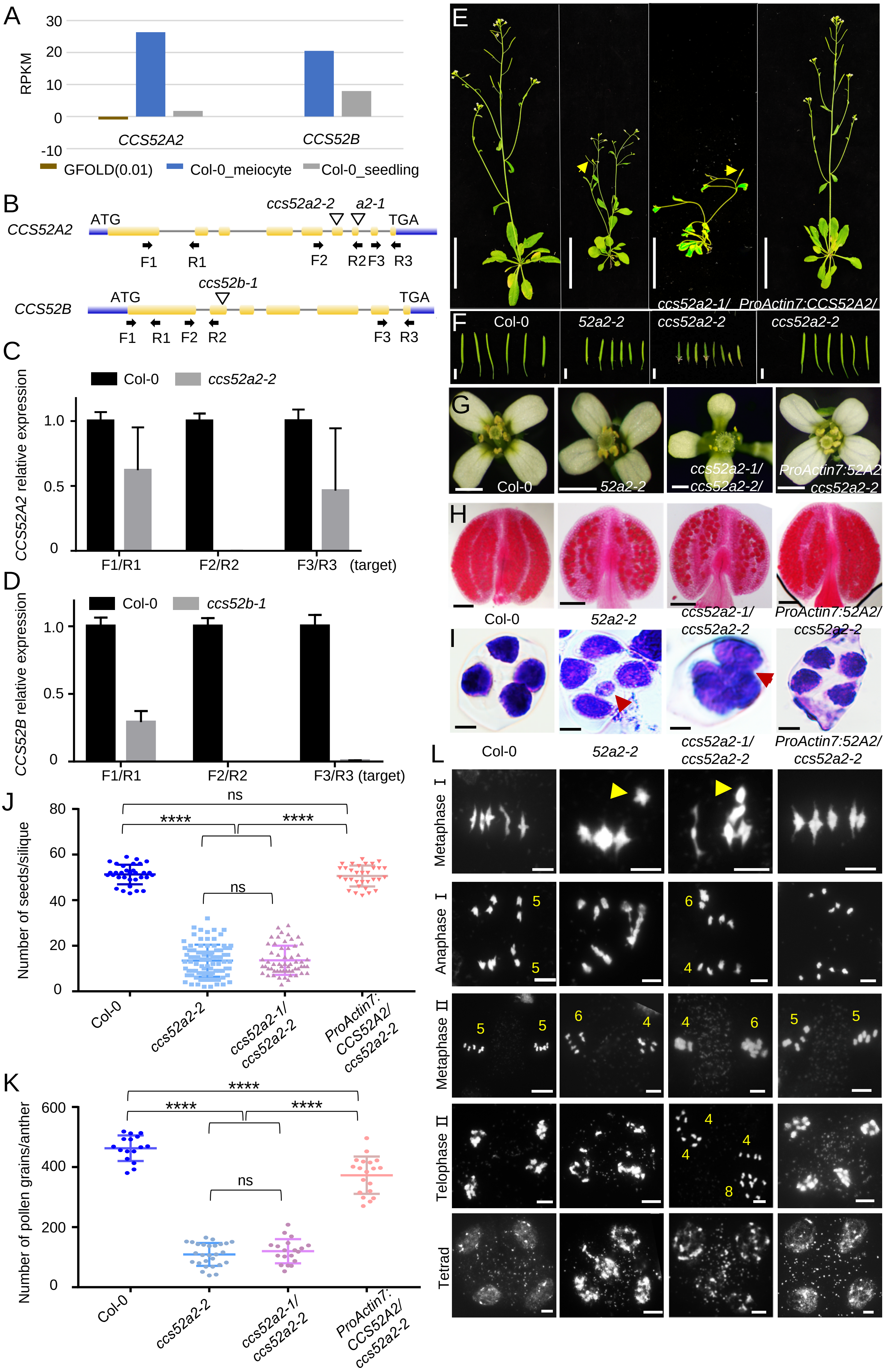

Supplementary Figure S7. *CCS52A2* is indispensable for vegetative development, fertility and meiosis. (Supports Figure 6).

(A) The relative expression level of *CCS52A2* and *CCS52B* in the previously published transcriptome data of male meiocytes and seedlings in *Arabidopsis* Col-0 (300 meiocyte masses (equal to 12,000 to 15,000 cells) from anthers and seedlings of 10 to 15 plants) using mRNA sequencing (Huang et al., 2019).

(B) Schematic representation of *CCS52A2*/*B* with T-DNA insertions (inverted triangles) in *ccs52a2* and *ccs52b.* Exons are shown as boxes. Arrows indicate primer positions.

(C) to (D) RT-qPCR amplification of the truncated transcript from *ccs52* loci using 10 inflorescences from Col-0 and T-DNA insertional mutant alleles *ccs52a2-2, ccs52b-1* using different primers as shown in (B). Data are presented as means ± SD.

(E) to (I) Plant growth phenotypes (E), siliques (F), flowers (G), pollen grains stained with Alexander Red (H) and carbol-fuchsin stained tetrad stage microspores (I) of Col-0, *ccs52a2-2*, *ccs52a2-1/ccs52a2-2* and *ProActin7:CCS52A2/ccs52a2-2* plants. Three independent lines of overexpressing *CCS52A2* transgenic allele showed similar results. Yellow arrowheads indicate short siliques in (E), red arrowheads indicate polyads in (I). Scale bar, 5 cm (E); 2 mm (F); 1 mm (G); 50 μm (H); 5 μm (I) respectively.

(J) Quantification of seed number per silique in Col-0, *ccs52a2*, *ccs52a2-1/ccs52a2-2* and *ProActin7:CCS52A2/ccs52a2-2* plants. For seed number analysis, 32 siliques from Col-0, 23 siliques from *ccs52a2-2*, 10 siliques from *ccs52a2-1/ccs52a2-2* and 32 siliques from *ProActin7:CCS52A2/ccs52a2-2* isolated from more than three independent plants were observed.

(K) The average number of pollen grains in Col-0, *ccs52a2-2*, *ccs52a2-1/ccs52a2-2* and *ProActin7:CCS52A2/ccs52a2-2* plants. 16 anthers for Col-0, 27 anthers for *ccs52a2-2*, 19 anthers for *ccs52a2-1/ccs52a2-2* and 19 anthers for *ProActin7:CCS52A2/ccs52a2-2* isolated from more than three independent plants were observed.

(L) Male meiotic chromosome behavior of Col-0, *ccs52a2-2*, *ccs52a2-1/ccs52a2-2* and *ProActin7:CCS52A2/ccs52a2-2* plants. For each meiotic stage in above-mentioned plants, more than 20 meiocytes isolated from more than three independent plants and three independent transgenic lines for transgenic plants were observed. Yellow triangles in metaphase Ⅰ show misaligned chromosomes in mutants. Yellow numbers indicate the observed unevenly distributed chromosome numbers. All pictures were taken with a ×100 objective using a fluorescence microscope (Zeiss Axio Imager). Scale bar, 5 μm.

The data in (C), (D), (J) and (K) are shown as means ± SD. The asterisks indicate significant differences assessed by two-tailed Student's *t* test (****p < 0.0001; ns. no significance). Source data are provided as a supplementary data set S1.

Supplementary Figure S8

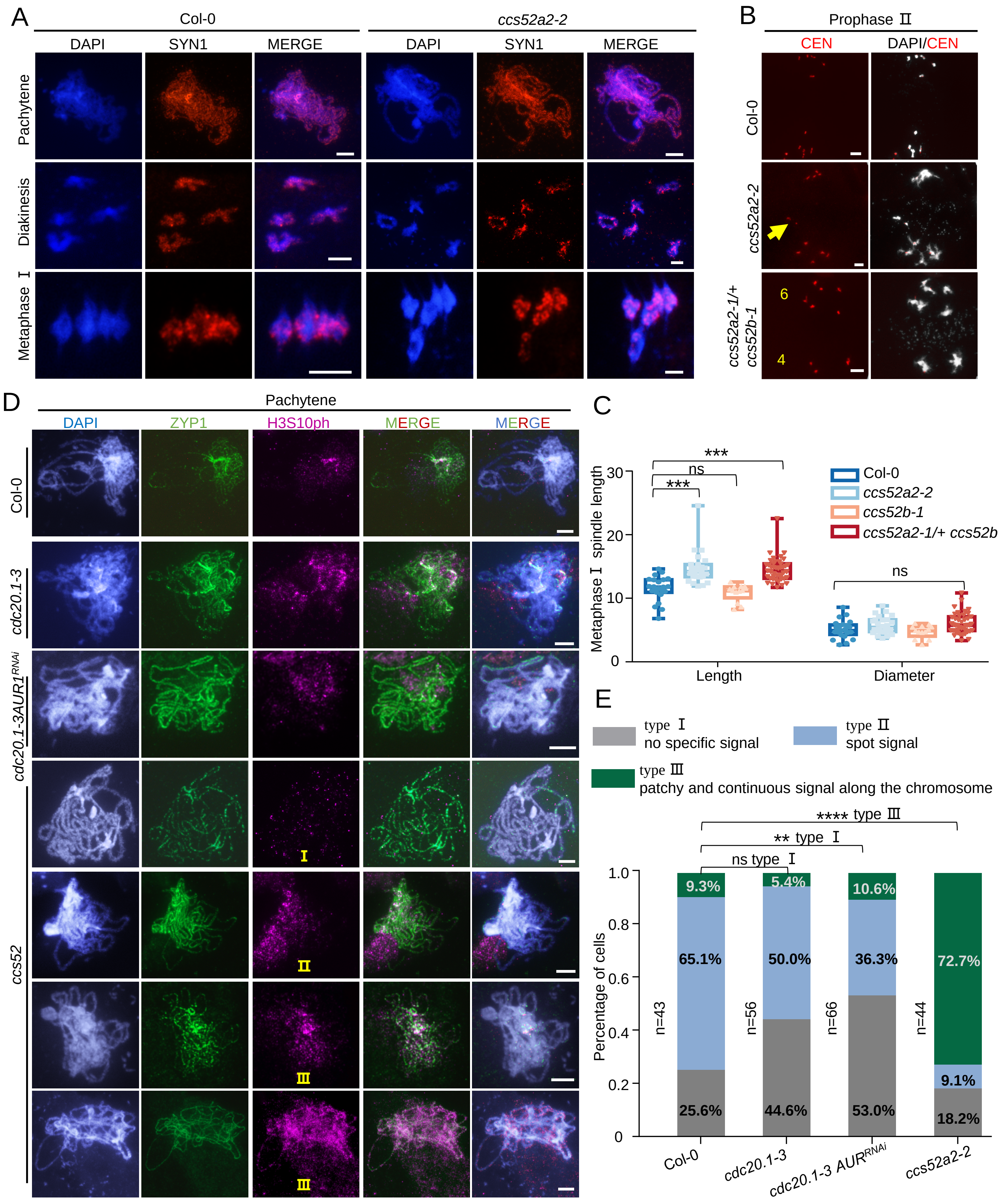

Supplementary Figure S8. CCS52A2 does not affect SYN1 loading and H3S10ph signal is enhanced in the *ccs52* mutant. (Supports Figure 6 and Figure 5F).

(A) Cohesin loading was unaffected in *ccs52a2-2*. Localization of cohesin SYN1 (red) with 4’, 6’-diamidino-2-phenylindole (DAPI) (blue) at pachytene, diakinesis and metaphase Ⅰ in Col-0 and *ccs52a2-2*. About 17 cells for each stage isolated from more than three independent plants were observed. Scale bar, 5 μm.

(B) Lagging chromosome were seen in *ccs52a2-2*. Meiotic chromosome behavior of Col-0, *ccs52a2-2*, *ccs52b-1*, and *ccs52a2-1/+ ccs52b-1* meiocytes at prophase Ⅱ. At least 15 cells for Col-0 and *ccs52a2* mutants isolated from more than three independent plants were observed. Yellow arrowhead indicates unusual centromere signal in a bivalent. Yellow numbers indicate the observed unevenly distributed chromosome numbers. Scale bar, 5 μm.

(C) Quantification of spindle length and diameter in (Figure 6C) at metaphase Ⅰ in Col-0 (n = 23), *ccs52a2-2* (n = 23), *ccs52b-1* (n = 13) and *ccs52a2-1/+ ccs52b-1* (n = 43) (n means cells number from 15 plants).The data is shown as box and whiskers, whiskers: min to max, show all points. Center lines of the box plots mean median; box limits, upper and lower quartiles mean first quartile (Q1) and third quartile (Q3); whiskers include lower whisker and upper whisker, which related to 1.5x interquartile range (IQR), the default threshold used to define the boundary for outliers; and the points outside mean outliers that the anomalous value which maybe result from measurement inaccuracies or exceptional incidents. p value were calculated using a two-tailed Student’s *t* test (***p < 0.001; ns. no significance).

(D) Distribution of ZYP1 (green) and H3S10ph (red) in Col-0, *cdc20.1*, *cdc20.1-3 AUR1*^{RNAi}, and *ccs52* mutant in pachytene cells. Type Ⅰ (no specific signal), type Ⅱ (spot signal), type Ⅲ (continuous and patchy signals that partially or completely cover the meiotic chromosomes) anti-H3S10ph fluorescence signal were defined. The classifications are labeled on the pictures. ZYP1 is used as a control to indicate meiotic chromosomes at pachytene. Scale bar, 5 μm.

(E) Percentage of different types of H3S10ph signal in *cdc20.1* and *ccs52* mutant shown in (D). The numbers of cells observed and the ratios near the histogram are labeled to indicate the number of cells counted and the percentage of different types, respectively. The proportion of type Ⅲ in Col-0 and *ccs52* were compared using two-sided Fisher’s exact test (****p < 0.0001), the proportion of type Ⅰ in *cdc20.1 AUR1*^{RNAi}, and *cdc20.1* compared with Col-0 using two-sided Fisher’s exact test (**p = 0.0056). Source data and statistic analysis of S8C are provided as a supplementary data set S1.

Supplementary Figure S9

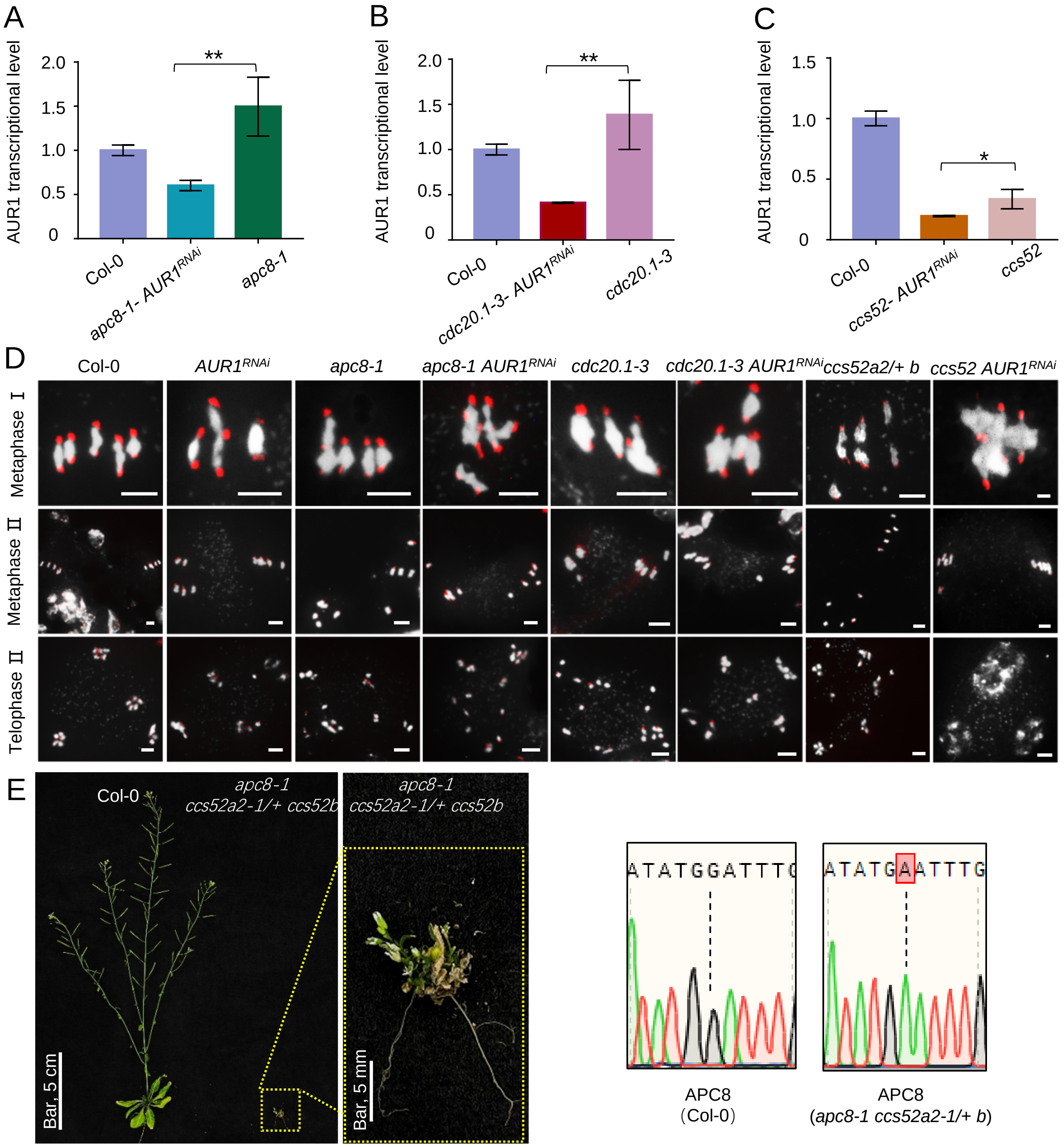

Supplementary Figure S9. **Genetic analyses of *APC8*, coactivator *CCS52/CDC20.1* and *AUR1* during meiotic chromosome segregation. (Supports supplementary Figure S7, Figure 1 and 6).**

(A) to (C) RT-qPCR analysis of the *AUR1* expression in inflorescences of Col-0, *apc8-1 AUR1^{RNAi}* (A), *cdc20.1-3 AUR1^{RNAi}* (B) and *ccs52 AUR1^{RNAi}* (C) with each single mutant *apc8-1*, *cdc20.1-3* and *ccs52,* respectively. The inflorescences were isolated from three independent plants. Statistical analysis was calculated using two-tailed Student's *t* test. *$p < 0.05$, **$p < 0.01$. The error bar represents the SD of each group.

(D) Meiotic chromosome phenotypes of Col-0*, cdc20.1-3, apc8-1, ccs52, AUR1^{RNAi}* and their higher-order mutants assayed by centromere FISH. *cdc20.1-3, apc8-1, ccs52* crossed with *AUR1^{RNAi}* to obtain their higher-order *cdc20.1-3 AUR1^{RNAi}, ccs52 AUR1^{RNAi}* and *apc8-1 AUR1^{RNAi}* mutants. For analyzing meiotic chromosomes behavior, 30 cells of *apc8, cdc20.1* and *AUR1^{RNAi}* at each meiotic stage, 78 cells for *apc8-1 AUR1^{RNAi}* Metaphase Ⅰ, 47 cells for *cdc20.1 AUR1^{RNAi}* Metaphase Ⅰ, 15 cells for *ccs52 AUR1^{RNAi}* Metaphase Ⅰ, 22 cells for *apc8-1 AUR1^{RNAi}* Metaphase Ⅱ, 45 cells for *cdc20.1 AUR1^{RNAi}* Metaphase Ⅱ, 15 cells for *ccs52 AUR1^{RNAi}* Metaphase Ⅱ, 12 cells for *apc8-1 AUR1^{RNAi}* Telophase Ⅱ, 55 cells for *cdc20.1 AUR1^{RNAi}* Telophase Ⅱ, 16 cells for *ccs52 AUR1^{RNAi}* Telophase Ⅱ, 25 cells for *apc8-1 AUR1^{RNAi}* tetrad, 44 cells for *cdc20.1 AUR1^{RNAi}* tetrad, 20 cells for *ccs52 AUR1^{RNAi}* tetrad isolated from more than three independent plants, were observed with similar meiotic defects at metaphase Ⅰ, metaphase Ⅱ, telophase Ⅱ and polyad except Col-0. Scale bar, 5 μm.

(E) Whole plant phenotypes of Col-0 and *apc8-1 ccs52a2-1/+ ccs52b.* The yellow dotted-lined rectangles magnifies *apc8-1 ccs52* mutants and shows vegetative growth defects. Scale bar, 5 cm, 5 mm. Screenshot of Sanger sequencing results of *APC8* in Col-0 and *apc8-1 ccs52* displayed in the right. Source data are provided as a supplementary data set S1.
